# Circulating biomarkers at diagnosis correlate with distant metastases of early luminal-like breast cancer

**DOI:** 10.1038/s41435-023-00220-z

**Published:** 2023-09-27

**Authors:** Yentl Lambrechts, Abhishek D. Garg, Giuseppe Floris, Kevin Punie, Patrick Neven, Ines Nevelsteen, Jannes Govaerts, François Richard, Annouschka Laenen, Christine Desmedt, Hans Wildiers, Sigrid Hatse

**Affiliations:** 1 Laboratory of Experimental Oncology (LEO), Department of Oncology, KU Leuven, Leuven, Belgium; 2 Laboratory of Cell Stress & Immunity (CSI), Department of Cellular and Molecular Medicine, KU Leuven, Leuven, Belgium; 3 Laboratory for Cell and Tissue Translational Research, Department of Imaging and Radiology, KU Leuven - Department of Pathology, University Hospitals Leuven, Leuven, Belgium; 4 Department of General Medical Oncology and Multidisciplinary Breast Center, University Hospitals Leuven, Leuven, Belgium; 5 Laboratory for Translational Breast Cancer Research (LTBCR), Department of Oncology, KU Leuven, Leuven, Belgium; 6 Leuven Biostatistics and Statistical Bioinformatics Center, KU Leuven, Leuven, Belgium

**Keywords:** Chemokines, Translational immunology

## Abstract

There is an urgent need for new and better biomarker modalities to estimate the risk of recurrence within the luminal-like breast cancer (BC) population. Molecular diagnostic tests used in the clinic lack accuracy in identifying patients with early luminal BC who are likely to develop metastases. This study provides proof of concept that various liquid biopsy read-outs could serve as valuable candidates to build a multi-modal biomarker model distinguishing, already at diagnosis, between early metastasizing and non-metastasizing patients. All these blood biomarkers (chemokines, microRNAs, leukemia inhibitory factor, osteopontin, and serum-induced functional myeloid signaling responses) can be measured in baseline plasma/serum samples and could be added to the existing prognostic factors to improve risk stratification and more patient-tailored treatment in early luminal BC.

## Introduction

Breast cancer (BC) is a heterogeneous disease, entailing several subtypes with distinct molecular patterns, histological features, and clinical outcomes. Among patients diagnosed with a hormone receptor-positive and epidermal growth factor receptor 2 (*HER2*) non-amplified BC (also referred to as ‘luminal-like’ BC subtype), approximately 20–30% will eventually develop distant relapse, leading to lethal disease. These recurrent tumors are mostly high-grade luminal tumors (‘luminal-like B’), which are more aggressive and associated with a poorer prognosis than the more indolent low-grade (‘luminal-like A’) tumors. Beyond clinicopathological risk factors, gene expression signatures like MammaPrint, Oncotype DX, EndoPredict, Prosigna, and Breast Cancer Index are used to estimate the likelihood of recurrence [1–6].

Unfortunately, more than 5% of patients classified as ‘low-risk’ by these gene expression signatures metastasize within five years, while most (70%) of ‘high-risk’ patients never develop metastatic disease [7]. Therefore, an urgent need exists for new and better tools to predict which patients with early luminal BC are more likely to develop metastases. Next to the evolving field of ctDNA in early BC, there is also potential to look at other circulating (plasma or serum) biomarkers. These biomarkers could be good candidates to be implemented as prognostic biomarkers in daily clinical practice since they are easily accessible by minimally invasive procedures. Although the exact biological mechanisms of the distant metastatic process are still largely unknown, many biological factors have been linked to the development of metastatic disease, and several of these factors are also circulating in the blood. For instance, micro-RNAs (miRNAs) are small single-stranded, non-coding RNAs regulating gene expression at the post-transcriptional level. Many miRNAs have been reported to play a regulatory role in metastasis and have therefore been named ‘metastamiRs’ [8, 9]. These can promote or inhibit metastases through various mechanisms, including regulation of tumor cell migration, invasion, colonization, cancer stem-cell properties, epithelial-mesenchymal transition, and microenvironment modulation [9–11]. Secondly, chemokines are chemotactic cytokines regulating the migration of immune cells between tissues and the interaction of cells within the tissue. Numerous studies have suggested that chemokines may also be essential in tumor growth, progression, and metastasis [12–14].

Osteopontin (OPN), secreted phosphoprotein 1 (SPP1), is a multifunctional acidic glycophosphoprotein in many tissues, including the breast. It is linked to matrix remodeling and regulates the immune response at multiple levels by influencing the secretion of interleukins, interferon-γ, and nuclear factor kappa B (NFkB) [15]. Interestingly, OPN has been associated with many metastasis-related mechanisms, such as cell proliferation, invasion, and tumor progression [16, 17]. Leukemia inhibitory factor (LIF) is a versatile cytokine with multiple functions in diverse cells/tissues by activating various signaling pathways. Not surprisingly, overexpression of LIF is also associated with metastasis-related processes, such as invasion and migration of cancer cells in BC [18, 19].

Immunological pathways, particularly pro- and anti-inflammatory cytokine networks, are also believed to play a dynamic role in the metastatic cascade [20]. Prognostic relevance of various serum-associated cytokines and specific immune cell phenotypes has been reported in patients with cancer. Notably, the ‘nuclear factor kappa light chain enhancer of activated B cells’ (NFκB) pathway and the interferon (IFN)-stimulated genes (ISG)/IFN pathway are recognized as the two broadest inflammatory pathways modulating cancer immunity as well as metastatic risk. A recently developed first-in-class serum functional immunodynamic status (sFIS) assay allows in vitro/ex vivo assessment of serum-induced myeloid NFκB and IFN/ISG response signaling to “mimic” the global in situ patient’s serum immune biology on the level of these two pathways [21]. Previously, such serum/plasma-induced myeloid IFN/ISG responses were associated with increased survival in ovarian cancer [22] and lung cancer [23] patients. On the other hand, serum-induced myeloid NFκB responses are associated with shortened survival and increased risk of metastasis in ovarian cancer patients [22].

The present study aimed to explore the potential metastasis-predicting capacity of the above-described circulating biomarkers by comparing the baseline plasma/serum profile between early luminal BC patients who relapsed at distance within five years after initial diagnosis and pair-wise matched BC patients who remained disease-free for at least seven years after initial diagnosis and primary treatment.

## Material & methods

### Ethics statement

This study was performed in compliance with the Helsinki Declaration and national law. All patients included in this study gave written consent for future translational research. The collection of patient data and blood sampling was approved by the ethics committee of our institution (Ethics Committee Research University Hospitals/Catholic University Leuven; study number: S56919; approval number: ML10867; approval date: 12 January 2015).

### Patient population

Eligible patients were selected from our institutional clinicopathological database, established by the Leuven Multidisciplinary Breast Center of the University Hospitals Leuven (Belgium). Our database contains extensive patient and tumor characteristics and follow-up information with relapse and survival data. Since 2003, the database has been linked to a large blood biobank, collecting baseline plasma and serum samples from all newly diagnosed BC patients who gave written informed consent.

Selected patients met the following criteria: (i) newly diagnosed between May 2003 and May 2018 with early BC (stage I-III); (ii) treated by surgery (with clear surgical margins, performed at our institution) and a combination of (neo-)adjuvant chemotherapy, endocrine therapy and/or radiotherapy; (iii) grade 2 or 3 invasive breast carcinoma of non-specific type (IBC-NST) (other histological subtypes or grade I IBC-NST were not allowed); (iv) estrogen receptor (ER) positive and human epidermal growth factor receptor 2 (*HER2*) non-amplified tumors (ER positivity was defined as at least 1% of cells staining positive according to ASCO-CAP guidelines [24] and *HER2* non-amplified also defined according to ASCO-CAP 2018 guidelines [25]); (v) development of secondary metastatic disease at least six months after early BC diagnosis, and maximum five years after BC diagnosis; (vi) no prior invasive BC; (vii) availability of baseline serum and plasma sample, collected at first BC diagnosis (before any treatment had started).

After selecting patients with secondary metastases (META group) was completed, a 1:1 matching control group was established, consisting of patients who did not develop distant metastases within at least seven years follow-up after initial diagnosis and primary treatment (NON-META group). Patient matching was based on age, (neo-)adjuvant chemotherapy yes/no, tumor grade (grade 2 or grade 3), and tumor stage (I, IIA, IIB, IIIA, IIIB, IIIC). Clinical TNM was considered for the stage matching for patients who received neoadjuvant treatment. For patients in the adjuvant setting, the pathological TNM was used. Due to the strict selection criteria and matching for multiple parameters, stage could only sometimes be perfectly matched but only partially differed within patient pairs.

### Blood collection and biomarker assessments

Peripheral blood was routinely sampled at the first clinical consultation, before any treatment, in 5 mL Vacutainer® SST II Advance tubes (for serum collection) and 4 mL BD Vacutainer® EDTA K2E tubes (for plasma collection).

A broad panel of baseline plasma chemokines (fractalkine/CX3CL1, GROα/CXCL1, IP-10/CXCL10, TECK/CCL25, TARC/CCL17, IL-8/CXCL8, MCP-1/CCL2, ITAC/CXCL11, BCA-1/CXCL13, RANTES/CCL5, MIP-3b/CCL19, CTACK/CCL27, MIP-3α/CCL20, 6-Ckine/CCL21, and CXCL12/SDF-1), LIF, and OPN were measured using bead-based immunoassays (Aimplex®, Biosciences Inc, and LEGENDplex™, BioLegend®). Plasma and serum levels of 91 miRNAs (listed in Supplementary Table S1) were measured using SYBR Green RT-qPCR (Qiagen). The serum functional immunodynamics status (sFIS) assay measures the serum-induced IFN/ISG response and NFκB signaling in a THP1 human myeloid reporter cell line system (InvivoGen) via a previously validated methodology [21]. All biomarker assessment procedures are described in detail in Supplementary Methods S2.

### Statistical analysis

All statistical analyses to assess the differences in baseline circulating chemokines, miRNAs, OPN, LIF, IFN/ISG, and NFκB response levels between early-relapsing and non-relapsing BC patients were performed in SAS software (version 9.4 of the SAS System for Windows). All blood biomarkers were univariably analyzed pairwisely using the Wilcoxon signed-rank test (two-tailed). Multiple testing correction was done by applying a false discovery rate (FDR) of 0.05.

Subsequently, to the univariable analysis, a multivariable analysis was performed in search of a combination of plasma/serum biomarkers – i.e., a biomarker model - that can predict the development of later metastases already at the time of primary diagnosis. Logistic regression modeling was used with metastasis as a binary response variable. A forward stepwise model selection procedure was applied to build a multivariable model of independent predictors. A 5% significance level was adopted for biomarkers to enter or leave the model. To deal with missing data, dummy variables were constructed for all biomarkers, indicating the observation status of that biomarker for a subject [1 = observed, 0 = missing]. Systematically including dummy variables in the model in interaction with their corresponding biomarkers allowed us to estimate the slope of the biomarker based on the available observations without excluding cases with missing values. The C-index is used to quantify the discriminative value of the model. This index takes values between 0.5 and 1, where 0.5 indicates discrimination no better than chance, and 1 indicates perfect discrimination. To avoid over-optimism due to evaluating the model on the data used for building the model, we applied an internal validation using 5-fold cross-validation to obtain a more honest estimate of the discriminative value or C-index. The data set was randomly split into five equally large subsets. Each of these subsets serves as a validation set, while the remaining four sets were combined into five corresponding training sets. A model-building procedure was performed on the five training sets, providing risk estimates for the patients in the corresponding validation sets. In this way, one risk estimate (probability of metastasis) was obtained for each subject in the dataset, derived from a model built on data in which that patient did not occur. The internally validated C-index was obtained through logistic regression with metastasis as a binary outcome, and the cross-validation predicted risk as a predictor variable.

## Results

### Patient characteristics

In total, 102 patients with early luminal-like BC who relapsed within five years after initial diagnosis and treatment (META group) were included and compared (1:1 matched) to 102 early luminal-like BC patients who remained disease-free for at least seven years after initial diagnosis and primary treatment (non-META group). Both groups’ baseline patient and tumor characteristics are summarized in Table 1, showing good similarity between META and NON-META groups. Median age was 58 years for both groups. For the META group, the most frequent anatomical locations of secondary metastases were bone and liver at 74% and 55%, respectively.

**Table 1:** Patient and tumor characteristics at baseline, and anatomical location of metastases in the META cohort.

Variables	Statistics	META group	NON-META group
**Age at diagnosis**			
	N	102	102
	Median	58	58
	Range	[28; 90]	[31; 83]
**Grade of tumor**			
Grade 2	n/N (%)	38/102 (37%)	38/102 (37%)
Grade 3	n/N (%)	64/102 (63%)	64/102 (63%)
**Progesterone receptor status**			
Positive	n/N (%)	82/102 (80%)	91/102 (89%)
Negative	n/N (%)	19/102 (19%)	11/102 (11%)
Unknown	n/N (%)	1/102 (1%)	0/102 (0%)
**Histological subtype**			
IBC-NST^a^	n/N (%)	102/102 (100%)	102/102 (100%)
**Stage in adjuvant treated patients**			
I	n/N (%)	8/82 (10%)	9/81 (11%)
IIA	n/N (%)	25/82 (30%)	28/81 (35%)
IIB	n/N (%)	19/82 (23%)	19/81 (23%)
IIIA	n/N (%)	21/82 (26%)	21/81 (26%)
IIIB	n/N (%)	1/82 (1%)	0/81 (0%)
IIIC	n/N (%)	8/82 (10%)	4/81 (5%)
**Stage in neo-adjuvant treated patients**			
I	n/N (%)	0/20 (0%)	0/21 (0%)
IIA	n/N (%)	2/20 (10%)	5/21 (24%)
IIB	n/N (%)	5/20 (25%)	0/21 (0%)
IIIA	n/N (%)	2/20 (10%)	6/21 (28%)
IIIB	n/N (%)	4/20 (20%)	5/21 (24%)
IIIC	n/N (%)	7/20 (35%)	5/21 (24%)
**Treatment regimes**			
Adjuvant chemotherapy	n/N (%)	43/102 (42%)	53/102 (52%)
Neo-adjuvant chemotherapy	n/N (%)	16/102 (16%)	17/102 (17%)
No chemotherapy	n/N (%)	59/102 (58%)	49/102 (48%)
Radiotherapy	n/N (%)	92/102 (90%)	95/102 (93%)
Endocrine therapy	n/N (%)	99/102 (97%)	100/102 (98%)
Refusal of any form of therapy	n/N (%)	1/102 (1%)	0/102 (0%)
**Location of relapse**			
Brain	n/N (%)	7/102 (7%)	-
Abdominal (Non liver)	n/N (%)	5/102 (5%)	-
Liver	n/N (%)	56/102 (55%)	-
Cutaneous	n/N (%)	1/102 (1%)	-
Lung	n/N (%)	21/102 (21%)	-
Bone	n/N (%)	76/102 (75%)	-
Lymph nodes	n/N (%)	16/102 (16%)	-
Others	n/N (%)	10/102 (10%)	-

^a^invasive breast carcinoma of non-specific type.

### Univariable evaluation of baseline circulating biomarkers

#### Baseline plasma circulating chemokines were not different between relapsing *versus* non-relapsing patients

Some chemokines (i.e., IP-10/CXCL10, TARC/CCL17, RANTES/CCL5, and CTACK/CCL27) had *P*-values < 0.05 (see Supplementary Table S3). However, none of the 15 measured circulating chemokines differed significantly after FDR correction between the META (*n* = 102) and NON-META (*n* = 102) groups.

#### Baseline miRNA profiles significantly differed between patients who did or did not develop metastasis

**Fig. 1:**
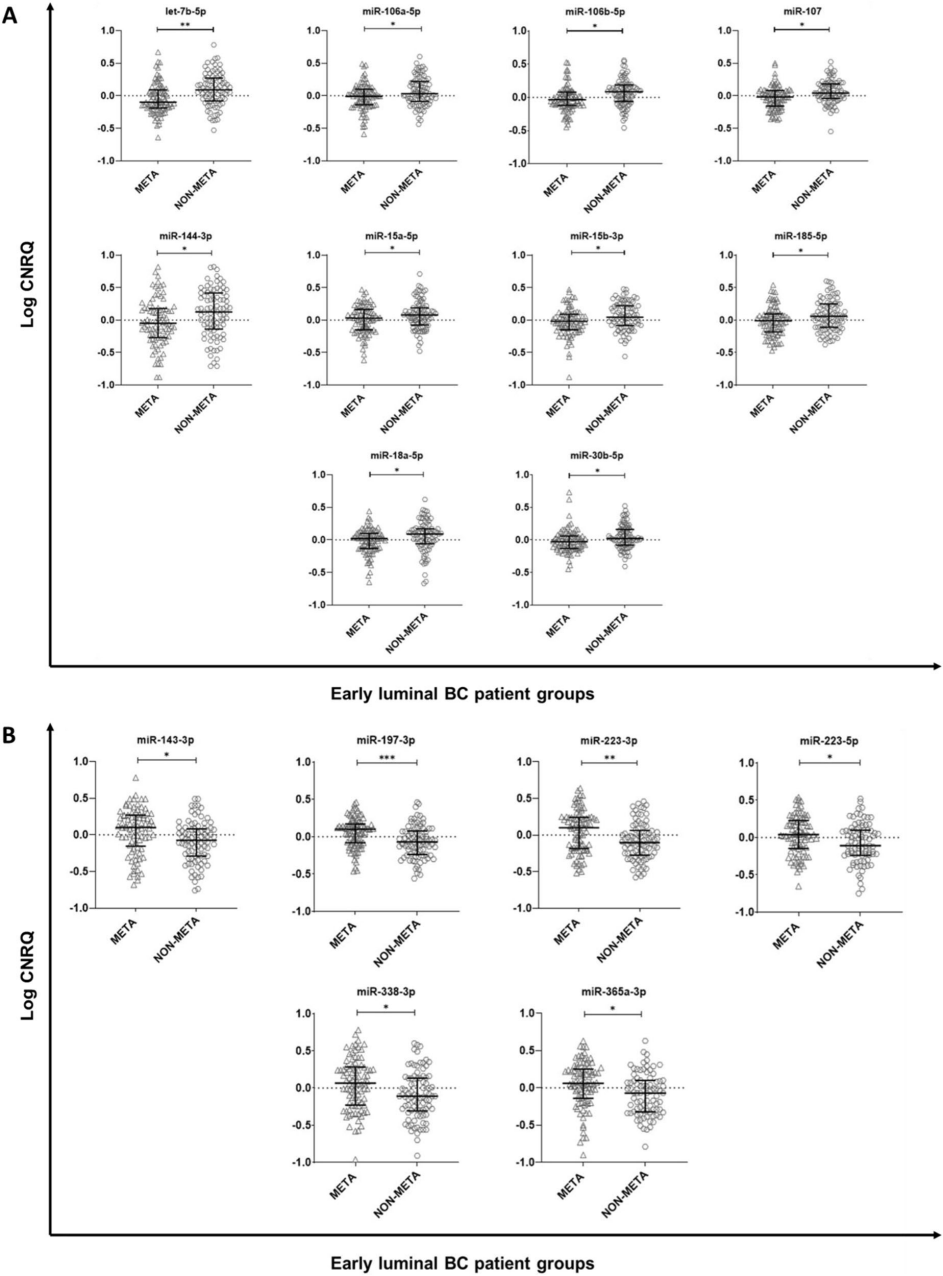
Differences in baseline circulating miRNAs profiles between early relapsed (META) versus non-relapsing (NON-META) luminal breast cancer patients. **A** Circulating miRNAs showing significantly decreased baseline expression in the early-relapsing (META) versus the non-relapsing (NON-META) group. **B** Circulating miRNAs showing significantly increased baseline levels in the early-relapsing (META) versus the non-relapsing (NON-META) group. The boxplots represent the IQR of the log10 CNRQ value of the miRNAs with outliers indicated as + in the META group and ○ in the NON-META group. The level of significance is indicated with * for FDR-corrected *P* ≤ 0.05, with ** for *P* ≤ 0.01, and with *** for *P* ≤ 0.001. The FDR-corrected *P*-values were calculated using a Wilcoxon signed-rank test. CNRQ Calibrated normalized relative quantity, IQR Interquartile range.

The 91-miRNA plasma/serum panel comprised multiple circulating miRNAs with significantly different baseline levels in non-relapsing *versus* relapsing patients. Most notably, after correction for multiple testing, baseline circulating miRNA levels of let-7b-5p (*P* = 0.006), miR-106a-5p (*P* = 0.045), miR-106b-5p (*P* = 0.015), miR-107 (*P* = 0.014), miR-144-3p (*P* = 0.015), miR-15a-5p (*P* = 0.045), miR-15b-3p (*P* = 0.032), miR-185-5p (*P* = 0.045), miR-18a-5p (*P* = 0.015), and miR-30b-5p (*P* = 0.045) were significantly lower, whereas baseline concentrations of circulating miR-143-3p (*P* = 0.015), miR-197-3p (*P* < 0.001), miR-223-3p (*P* = 0.006), miR-223-5p (*P* = 0.045), miR-338-3p (*P* = 0.045), and miR-365a-3p (*P* = 0.025), were significantly increased in relapsing (META, *n* = 88) *versus* non-relapsing (NON-META, *n* = 88) patients (Fig. 1). A complete list of the 91 miRNAs investigated in this study is available in Supplementary Table S1.

#### Baseline circulating serum-induced myeloid IFN/ISG response, but not NFκB signaling, significantly differed between relapsing and non-relapsing patients

**Fig. 2:**
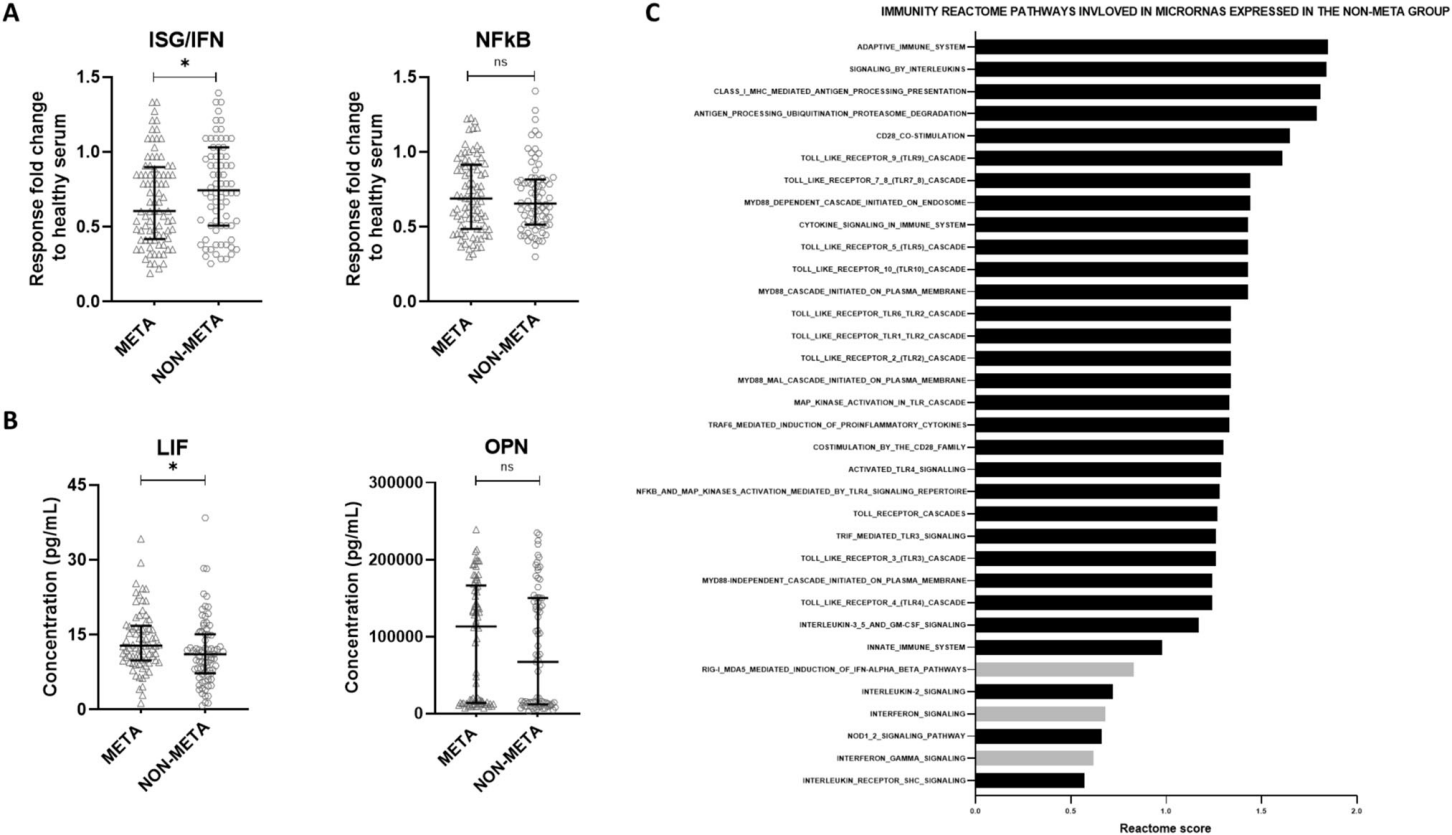
Baseline serum-induced NFkB and/or IFN/ISG response-signaling in the sFIS assay and baseline plasma levels of LIF and OPN in early-relapsing (META) versus non-relapsing (NON-META) luminal breast cancer patients. In addition, ranking summary of the immunity pathways analyzed by the REACTOME software. **A** Baseline serum-induced responses of human myeloid THP1 cells reporting for the IFN/ISG responses are significantly decreased in the early relapsing (META) versus the non-relapsing (NON-META) patients, whereas baseline serum-induced NFκB signaling responses showed no statistical difference between early-relapsing (META) versus non-relapsing (NON-META) patients. **B** Baseline plasma levels of LIF showed a significant increase in the early-relapsing (META) versus non-relapsing (NON-META) patients, whereas baseline plasma levels of OPN showed no statistically significant difference between the early-relapsing (META) versus non-relapsing (NON-META) patients. The boxplots represent the IQR of the response fold change data (compared to normal human serum) of the baseline serum samples (**A**) and the baseline plasma concentration (pg/mL) of circulating LIF and OPN (**B**). The outliers are indicated as + in the META group and ○ in the NON-META group. The level of significance is indicated with * for FDR-corrected *P* ≤ 0.05 and ns: no statistical significance. The FDR-corrected *P*-values were calculated using a paired Wilcoxon signed-rank test. **C** The represented pathways are analyzed from the miRNAs highly expressed in non-metastasizing luminal breast cancer patients. The Reactome score represents the alignment of multiple miRNAs involved in pathways in Homo Sapiens. The higher the score, the better the alignment of the multiple miRNAs with that particular pathway. Pathways that are involved in the interferon signaling are colored in gray (INTERFERON_GAMMA_SIGNALING, INTERFERON_SIGNALING, and RIG-I_MDA5_MEDIATED_INDUCTION_OF_IFN-ALPHA_BETA_PATHWAYS). IFN Interferon, IQR Interquartile range, ISG Interferon-stimulated genes, LIF Leukemia Inhibitory Factor, NFκB Nuclear Factor Kappa-light-chain enhance of activated B cells, OPN Osteopontin.

After correction for multiple testing, a significant decrease in the serum-induced myeloid IFN/ISG response levels (*P* = 0.023) was observed in the relapsing (META, *n* = 68) compared to the non-relapsing (NON-META, *n* = 68) patients (Fig. 2A). Median baseline fold change response levels were 0.61 (IQR: 0.41; 0.88) *versus* 0.77 (IQR: 0.44;1.03) for META and NON-META, respectively. On the other hand, median baseline serum-induced myeloid NFκB response was not significantly different in the relapsing (META) *versus* the non-relapsing (NON-META) patients. Baseline fold change response levels of NFκB response were 0.66 (IQR:0.50;0.91) *versus* 0.68 (IQR: 0.51; 0.83) for META and NON-META, respectively.

#### Baseline plasma levels of circulating LIF but not OPN significantly differed between relapsing and non-relapsing patients

A significantly higher baseline level of LIF (*P* = 0.006) was observed in the plasma samples of the relapsing (META, *n* = 66) group compared to the non-relapsing (NON-META, *n* = 66) group (Fig. 2B). Median plasma concentrations of LIF were 13.00 pg/mL (IQR: 10.00; 17.60) *versus* 10.85 pg/mL (IQR: 7.10; 14.90) for META and NON-META, respectively. This difference remained significant after correction for multiple testing (*P* = 0.012). In contrast, after correction for multiple testing, the two groups’ baseline circulating plasma levels of OPN showed no significant difference. Baseline median concentrations of OPN were 94 862 pg/mL (IQR: 12 837; 142 184) *versus* 72 339 pg/mL (IQR: 12 174; 149 976) for META and NON-META, respectively.

### Multi-modal model signature of seven biomarkers predicting secondary metastasis

**Fig. 3:**
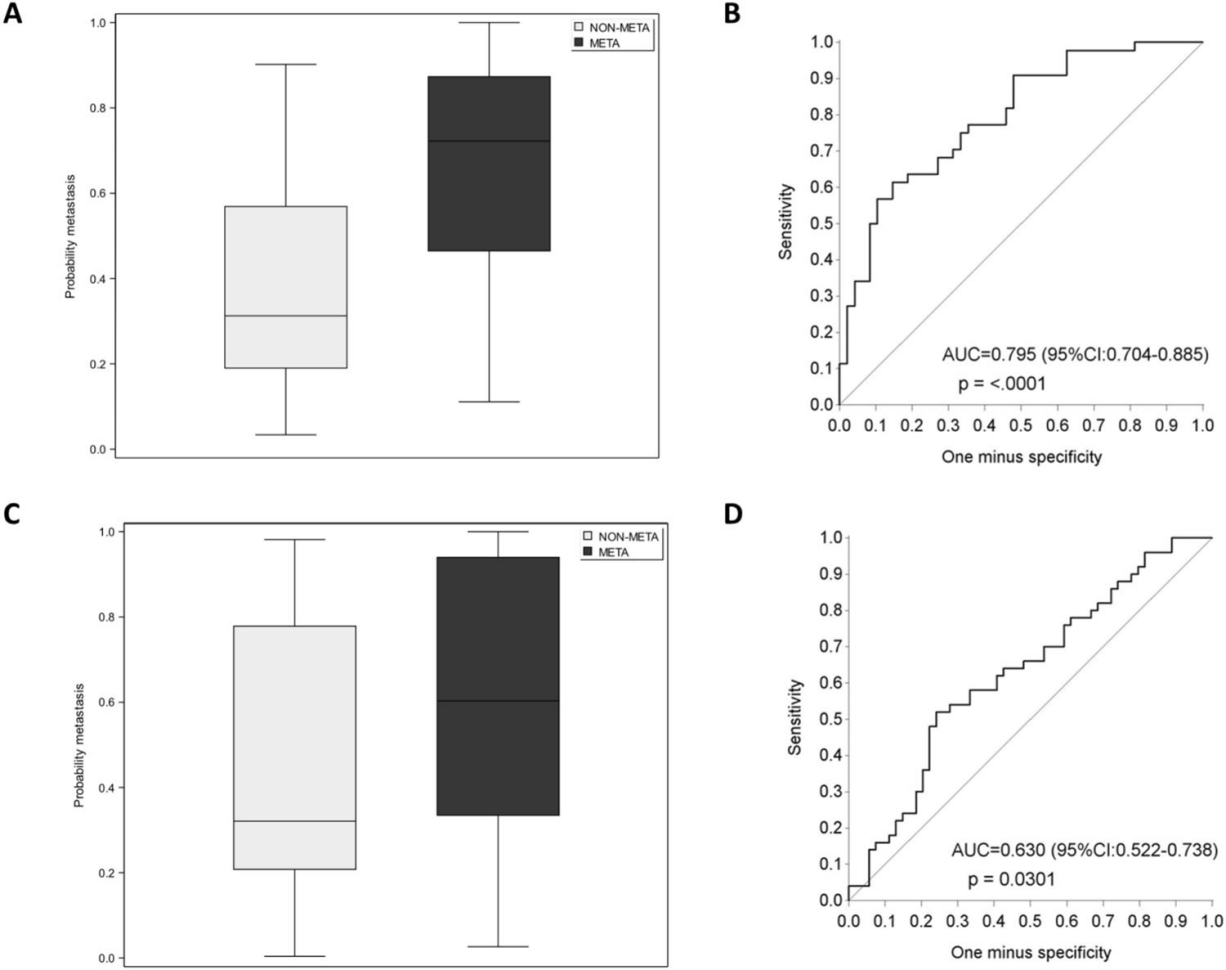
Multivariable logistic regression analysis (MVA) model predicting the probability of metastasis by group (early-relapsing versus non-relapsing). **A** Boxplot representing the probability of metastasis using the seven-biomarker combination including miR-197-3p, miR-139-5p, LIF, miR-106b-5p, serum-induced IFN/ISG response, miR-652-3p, and miR-133b. The model fairly well distinguishes the early-relapsing (META) from the non-relapsing (NON-META) group. **B** AUC/ROC curve of the MVA model illustrating the discriminative value of the model. **C** Boxplot representing the internally validated metastasis probability by using the seven-biomarker model. The model still distinguishes to some extent the early-relapsing (META) from the non-relapsing (NON-META) group. **D** AUC/ROC curve of the internally validated MVA model, illustrating the discriminative value of the model. The boxplots represent the interquartile range. AUC Area under the curve, CI Confidence interval.

In addition to the univariable analysis, we performed a multivariable logistic regression analysis (MVA) to search for an optimal combination of plasma/serum biomarkers – biomarker model – that can predict the development of later metastases at first diagnosis of early BC. The resulting multivariable model included seven biomarkers: miR-197-3p (*P* = 0.008), miR-139-5p (*P* < 0.001), LIF (*P* = 0.007), miR-106b-5p (*P* = 0.003), serum-induced IFN/ISG response (*P* = 0.006), miR-652-3p (*P* = 0.015), and miR-133b (*P* = 0.025) with independent prognostic value (Table 2). The model allowed fairly good discrimination between early-relapsing and non-relapsing patients, as demonstrated by the C-index (95% CI) of 0.79 (0.70:0.88) (Fig. 3A, B). However, the model’s discriminative value may be overestimated because the performance index (C-index) is obtained from the same data on which the model was built. To account for this, we applied an internal validation using 5-fold cross-validation to obtain a more conservative estimate of the discriminative value. This cross-validation resulted in a C-index of 0.63 (0.52:0.74), which still suggests a moderate discriminative value for the model (Fig. 3C, D).

**Table 2:** Predictive model of seven biomarkers discriminating between early-relapsing (META group) and non-relapsing (NON-META group) luminal breast cancer patients at the moment of diagnosis.

Variable	Parameter estimate (beta)	Units	Odds Ratio (95% CI)	*P*-value
Intercept	0.35	.		.
miR-197-3p	3.49	0.1	1.42 (1.16;1.74)	< 0.001
miR-139-5p	−3.20	0.1	0.73 (0.62;0.85)	< 0.001
Leukemia inhibitory factor	0.08	1	1.09 (1.02;1.16)	0.007
miR-106b-5p	−3.15	0.1	0.73 (0.59;0.90)	0.003
Serum-induced IFN/ISG response	−1.69	1	0.18 (0.05;0.62)	0.006
miR-652-3p	−2.61	0.1	0.77 (0.62;0.95)	0.015
miR-133b	−1.39	1	0.25 (0.07;0.84)	0.025

Odds ratio with 95% CI and *P*-values are presented for a x-units increase of the biomarker. Negative parameter estimate values and odds ratio < 1 indicate lower probability of metastasis with increasing biomarker values. Positive parameter estimates and odds ratio > 1 indicate higher probability of metastasis with increasing biomarker values.

*CI* Confidence interval.

Model C-index (95% CI): 0.795 (0.704:0.885).

### Pathway analysis reveals that miRNAs which are more expressed in long-term non-metastasizing patients are associated with IFN signaling

Above results with miRNA and the IFN/ISG response in the sFIS assay in the NON-META group made us curious whether miRNAs and IFN signaling are intertwined in the early luminal BC setting. Therefore, we examined if miRNAs, which are more expressed in the NON-META group and thus are associated with a lower metastasis risk, also impact IFN signaling, thereby explaining their co-enrichment with induction of IFN/ISG response in the sFIS assay. Unbiased pathway analyses were performed with the REACTOME pathway database for two miRNA expression categories: miRNAs overexpressed in the NON-META group and miRNAs overexpressed in the META group. We found that miRNAs overexpressed in the NON-META group are associated with many (*n* = 34) immunity-related pathways. Furthermore, these miRNAs were linked with pathways representing IFN signaling, i.e., type I IFN or IFNG signaling pathways (Fig. 2C). Conversely, miRNAs overexpressed in the META group showed no association with IFN-related pathways. A detailed list of all the pathways associated with miRNAs expressed in the NON-META and META groups can be found in Supplementary Tables S4 and S5, respectively. Altogether, this emphasizes that elevated IFN/ISG signaling in the serum is a dominant characteristic of the NON-META group and a point of convergence for miRNA and immune biology.

## Discussion

**Fig. 4:**
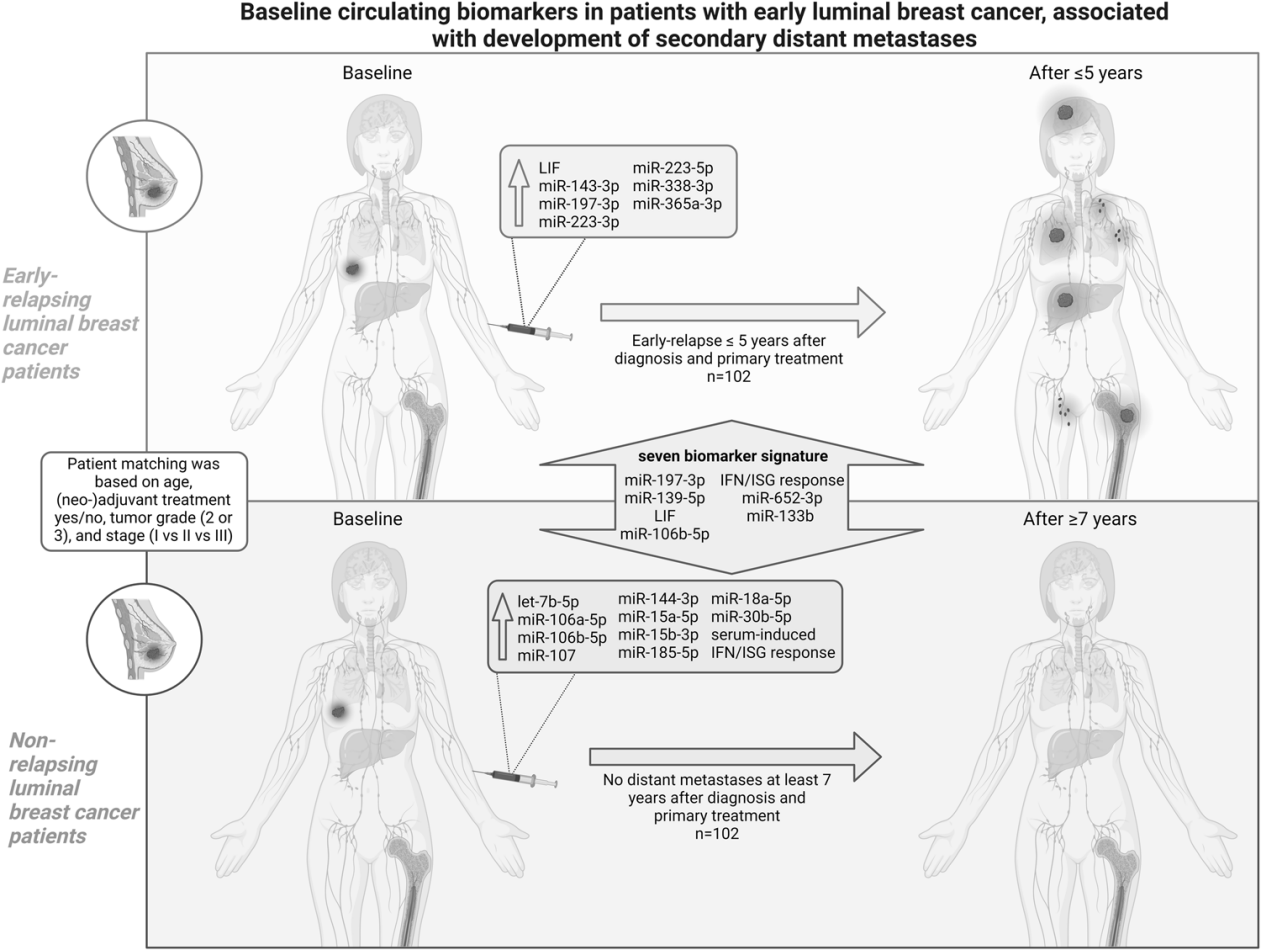
Overview of the evaluated candidate prognostic biomarkers of early recurrence based on our study results. Non-relapsing luminal breast cancer patients remained disease-free for at least seven years after primary diagnosis and treatment. These patients exhibit a profile of higher plasma/serum levels of let-7b-5p, miR-106a-5p, miR-106b-5p, miR-107, miR-144-3p, miR-15a-5p, miR-15b-3p, miR-185-5p, miR-18a-5p, miR-30b-5p, and serum-induced IFN/ISG response at diagnosis. Early-relapsing luminal breast cancer patients developed distant metastases within five years after primary diagnosis and treatment. These patients exhibit a profile of higher plasma/serum levels of miR-143-3p, miR-197-3p, miR-223-3p, miR-223-5p, miR-338-3p, miR-365a-3p, and LIF at diagnosis. In addition, the seven-biomarker signature, which holds miR-197-3p, miR-139-5p, LIF, miR-106b-5p, serum-induced IFN/ISG response, miR-652-3p, and miR-133b, showed substantial predictive value in the early luminal breast cancer setting for future development of metastatic disease. IFN Interferon, ISG Interferon-stimulated genes, LIF Leukemia inhibitory factor.

This study explored the potential metastasis-predicting capacity of various circulating plasma/serum biomarkers (chemokines, miRNAs, OPN, and LIF), as well as functional immuno-dynamic status (serum-induced myeloid IFN/ISG and NFκB signaling response), which all have been previously reported to be involved in metastatic processes [9–19]. To this end, baseline expression values of the different blood biomarkers were compared between patients with *HER2*-non-amplified luminal-like BC who developed distant metastases within five years and patients who remained disease-free for at least seven years after initial diagnosis and primary treatment. Our findings indicate that significant differences in circulating biomarkers of relapsing *versus* non-relapsing patients can already be detected during primary BC diagnosis. Fig. 4 summarizes the baseline circulating biomarkers for which a significant relation with development of secondary metastases was found in our study.

MiRNAs and their interplay in the metastatic scene have been extensively described in the literature [9–11]. However, the precise role of many miRNAs as tumor suppressors or oncogenic drivers is often contradicting in different publications. Our results suggest that miR-143-3p, miR-197-3p, miR-223-3p, miR-223-5p, miR-338-3p, and miR-365a-3p are positively correlated with metastasis, whereas miRNAs let-7b-5p, miR-106a-5p, miR-106b-5p, miR-107, miR-133b, miR-139-5p, miR-144-3p, miR-15a-5p, miR-15b-3p, miR-185-5p, miR-18a-5p, miR-30b-5p, and miR-652-3p may show a negative association with metastasis in the luminal BC setting. Many of these findings could correlate with earlier reports concerning the functionality of these miRNAs regarding metastasis in different cancer types [8, 11, 26–35]. Most of these reports describe the role of miRNAs at the (breast) tumor level, while information on circulating miRNAs is scarce. Besides, studies that did examine circulating miRNAs primarily compared blood samples from BC patients with samples from healthy controls [36]. Few other studies have reported on circulating miRNAs, measured at primary BC diagnosis and discriminating between future relapsing and non-relapsing patients. Elghoroury and colleagues investigated the clinical utility of let-7 as a prognostic biomarker for BC. They found that let-7 was negatively correlated with metastasis in BC patients [37], which is consistent with our results. It should be noted, however, that previous studies did not specifically investigate circulating miRNA profiles in luminal-like BC as we did in our study. Since different BC subtypes are largely distinct disease entities with divergent underlying biological/molecular driving mechanisms, specific miRNAs likely have opposite effects in luminal-like and hormone receptor-negative or *HER2*-amplified subtypes.

Another baseline circulating biomarker that was significantly elevated in relapsing patients in our study was LIF, which is known in the literature as an oncogenic driver. For instance, Li et al. described that LIF overexpression promotes invasion and migration of BC cells in vitro and metastasis in vivo [18]. Moreover, Shin et al. and Viswanadhapalli et al. concluded that LIF contributes to tumor cell proliferation and metastases formation via the autocrine and paracrine pathways in primary breast tumors [19, 38]. Interestingly, there seems to be a relationship between LIF and miR-197-3p, which was the most significantly altered (upregulated in relapsing patients) miRNA in our study. Xu et al. demonstrated that LIF receptor antisense RNA1 (LIFR-ASR1), a novel cancer-related long codon RNA transcribed from the *LIFR* gene in an antisense manner, is under-expressed in BC. Low expression of LIFR-ASR1 is associated with poor prognosis, while overexpression inhibits BC cell proliferation, colony formation, migration, and invasion. However, these tumor-inhibitory effects of LIFR-AS1 are abolished by miR-197-3p through interaction with LIFR-AS1 [39]. Notably, miR-197-3p happens to exert such tumor-promoting function in a variety of cancer types. To our knowledge, LIF has never been described before as having a role in the metastasis process of luminal BC. Our results suggest that baseline circulating LIF is a potential metastasis driver in luminal-like BC patients, but external validation is required here.

Given the established connection between inflammation and cancer and the well-documented immunological involvement in controlling tumor development and progression [20], circulating cytokines and other immune modulators could also be proposed as interesting candidate prognostic biomarkers in cancer patients. However, individual cytokines are often not specific enough to serve as a unique, reliable biomarker in clinical practice. Moreover, the quantity measured in serum for a given immune-related factor is not always linearly associated with its actual biological functions because immuno-stimulatory and immuno-suppressive factors coexist in full serum derived from cancer patients and may counteract each other. Thus, quantitative measurements of individual proteins cannot capture the functionally “integrated immunological activity” in the blood. This could also be valid when considering the negative results of the comparative plasma chemokine measurements discussed above. To address this issue, we used a recently established method [21]. The global in situ serum ‘immunome’ can be appraised by assessing the in vitro response of myeloid reporter cells to serum-induced NFκB and/or IFN/ISG pathway signaling (sFIS assay). In our exploratory study, the IFN/ISG pathway response levels were significantly lower in serum samples from metastasizing *versus* non-metastasizing patients. The IFN/ISG pathway and its relation to BC have been addressed in several studies, where type I IFN or IFN response is positively correlated with outcome, including in circulation. Furthermore, it has been shown that BC patients harboring a higher expression of the IFN signature genes have better overall survival [40–46]. In line with our findings, Garg et al. found that IFN is positively correlated with improved survival in ovarian and breast malignancies [47]. This positive correlation between IFN/ISG activity level and outcome was also confirmed in our multivariable logistic regression analysis, where serum-induced IFN/ISG response levels, in combination with the six other circulating biomarkers miR-197-3p, miR-139-5p, LIF, miR-106b-5p, miR-652-3p, and miR-133b, constituted the biomarker combination affording the best metastasis prognostic capacity. The model reported a negative parameter estimate with an odds ratio below 1 for serum-induced IFN/ISG response, indicating that the higher the serum level of this biomarker, the lower the probability of metastasis occurrence. Higher serum-induced IFN/ISG response levels thus seem to be associated with suppression of distant metastases, possibly via the involvement of IFNγ in cancer immune surveillance [48]. Furthermore, there appears to exist an association between miRNAs that predict a low metastatic risk and IFN/ISG signaling from the sFIS assay. Using an unbiased pathway analysis, we found that miRNAs overexpressed in the NON-META group were significantly associated with regulating IFN signaling pathways. This suggests that these miRNAs are biologically connected to sFIS assays’ IFN/ISG response readout and that the association is set in a pathway-dependent manner for IFN signaling.

A limitation of our study is the relatively small sample size, mainly because we used strict selection and matching criteria. In addition, sufficiently large amounts of sample material were required to perform all the different biomarker analyses, which resulted in fewer available matched patient pairs for some biomarkers. Therefore, the results should be interpreted with caution and still need to be confirmed in external validation cohorts. On the other hand, the highly homogenous cohort achieved by the very stringent selection process represents a major strength of the study. Also, genomic information was provided for only some BC patients, which gives unclarity whether the groups were prognostically balanced according to gene expression signatures. Since longer follow-up was unavailable for all patients from the non-relapsing group, the minimum follow-up period was only seven years. We cannot ensure these patients remain disease-free in the long term (beyond seven years). Therefore, our results only indicate the prediction capacity in luminal-like BC patients’ “early” distant relapse setting.

A small proportion of patient pairs in our study cohort (16%) were treated with first-line neoadjuvant chemotherapy, while the majority received first-line surgery. As stipulated before, the strict matching criteria applied to select the study cohort resulted in a limited number of eligible patient pairs. In order to maximally increase the cohort size, all available patient pairs were included, whether or not they received neoadjuvant chemotherapy. It would be interesting to investigate whether the prognostic capacity of the evaluated circulating biomarkers is comparable between these two treatment groups. Unfortunately, the number of matched patient pairs with neoadjuvant treatment was insufficient to perform such sub-analyses. Therefore, further validation in larger cohorts of patients with early hormone receptor-positive BC is definitely required.

To carry out this study, we used baseline samples from our biobanking project, initiated in 2003 at the University Hospitals Leuven, and applying standardized baseline sample collection procedures for all consenting women newly diagnosed with early BC at our institution. This BC blood biobank is connected with an extensive clinical database, which includes detailed clinicopathological information and follow-up data. This combination of a comprehensive database and blood bank is unique and makes it possible to carry out this biomarker study. Importantly, the identified prognostic biomarkers were detected in the plasma/serum of the patient. Liquid biopsy biomarkers are promising as they are easily accessible via minimally invasive procedures, and can be easily repeated during the disease course.

This study was the first study to analyze in a concerted manner a large diversity of circulating biomarkers related to metastasis in baseline plasma/serum samples within an early luminal BC population. Our data strongly suggest circulating miRNAs such as let-7b-5p, miR-106a-5p, miR-106b-5p, miR-107, miR-144-3p, miR-15a-5p, miR-15b-3p, miR-185-5p, miR-18a-5p, and miR-30b-5p, as well as serum-induced IFN/ISG response levels at baseline are inversely correlated with subsequent distant metastases. Other baseline circulating miRNAs such as miR-143-3p, miR-197-3p, miR-223-3p, miR-223-5p, miR-338-3p, and miR-652-3p, as well as plasma LIF levels, are significantly higher in patients who develop secondary metastatic disease. We propose a seven-biomarker baseline plasma/serum model that moderately predicts the development of later metastases in early luminal BC. It should be stressed, however, that this is an exploratory study, involving relatively low patient numbers. The results certainly need to be validated in larger independent cohorts before any clinical applicability could possibly be envisioned. Even when externally validated, this ‘model’ will still be far from perfect and not yet ready for further clinical development. Also, the biological role of the baseline biomarkers constituting the predictive model and their precise link to the future development of metastatic disease needs to be elucidated. Nevertheless, our results provide proof of concept for baseline blood biomarkers as additional prognostic tools to help estimate the risk of recurrence in patients with hormone receptor-positive BC, where treatment decisions are often difficult. External validation on a large, independent cohort is required to confirm our findings. Also, further research is needed to evaluate whether the studied factors could improve prognostication when combined with classic clinicopathological factors and molecular tests, as it seems unlikely that these circulating biomarkers will suffice by themselves to predict secondary metastases reliably. Future studies will assess whether these variations in blood biomarkers, measured at diagnosis, also mirror baseline differences in the tumor immunological microenvironment between patients who will or will not develop secondary metastasis later in time. For this purpose, core needle biopsies collected at diagnosis can be used. As previously indicated, pro- and anti-inflammatory pathways are believed to be involved in the metastatic process. Thus, evaluation of interferons, cytokines, and chemokines at the tumor level could indicate whether these inflammatory pathways are already altered in the tumor at the time of diagnosis and whether the differences in the blood compartment of relapsing *versus* non-relapsing patients are caused by the evolving tumor. This could further corroborate our conclusion that these circulating biomarkers may have potential for estimating already at diagnosis the likelihood of subsequent metastasis. Besides their prognostic potential for upfront prediction of secondary metastases, these biomarkers could be explored as candidate new therapeutic targets for preventing metastatic disease.

### Supplementary information

Supplementary Table S1: Baseline circulating miRNA levels measured in the early-relapsing (META) versus non-relapsing (NON-META) patients.

Supplementary Methods S2

Supplementary Table S3: Baseline plasma circulating chemokine differences between the early-relapsing (META) versus non-relapsing (NON-META) patients

Supplementary Table S4: Pathway ranking summary of the pathway analysis from the REACTOME software.

Supplementary Table S5: Pathway ranking summary of the pathway analysis from the REACTOME software.

## Data Availability

The datasets used and/or analyzed during the current study are available from the corresponding author on reasonable request.
