# Supplementary material for: Circulating biomarkers at diagnosis correlate with distant metastases of early luminal-like breast cancer
Source: Genes Immun. 2023 Sep 27;24(5):270–9. doi: 10.1038/s41435-023-00220-z (PMC10575765; doi:10.1038/s41435-023-00220-z)
Supplement: Supplementary file 1 — Supplementary Table S1: Baseline circulating miRNA levels measured in the early-relapsing (META) versus non-relapsing (NON-META) patients. [file 41435_2023_220_MOESM1_ESM.docx]

**Supplementary Table S1**

*Supplementary Table S1: Baseline circulating miRNA levels measured in the early-relapsing (META) versus non-relapsing (NON-META) patients. The number of patients included in each miRNA analysis, median, interquartile range (IQR), raw P-value, and FDR-corrected P-value are reported. Median and IQR values are reported as logarithmic (log10) transformed calibrated normalized relative quantities (CNRQ) values. The P-values were calculated using a paired Wilcoxon signed-rank test. The P-values that were statistically significant are reported in italics. FDR: false discovery rate.*

|  | **NON-META** | | **META** | | ***P*-values** | |
| --- | --- | --- | --- | --- | --- | --- |
| **Biomarker** | **N** | **Median [IQR]** | **N** | **Median [IQR]** | **Raw** | **FDR** |
| let-7a-5p | 85 | 0.03 [-0.14;0.22] | 85 | 0.01 [-0.13;0.14] | 0.231 | 0.329 |
| let-7b-5p | 86 | 0.09 [-0.08;0.27] | 86 | -0.10 [-0.18;0.11] | *<0.001* | *0.006* |
| let-7d-3p | 88 | -0.02 [-0.16;0.13] | 88 | -0.02 [-0.11;0.10] | 0.798 | 0.844 |
| let-7d-5p | 84 | 0.01 [-0.12;0.13] | 84 | 0.01 [-0.15;0.14] | 0.543 | 0.660 |
| let-7f-5p | 89 | 0.04 [-0.07;0.21] | 89 | 0.01 [-0.12;0.10] | 0.112 | 0.209 |
| let-7g-5p | 89 | 0.03 [-0.09;0.14] | 89 | -0.01 [-0.09;0.09] | 0.107 | 0.209 |
| let-7i-5p | 86 | 0.08 [-0.06;0.19] | 86 | -0.02 [-0.11;0.09] | 0.059 | 0.149 |
| miR-101-3p | 86 | 0.14 [-0.08;0.31] | 86 | 0.01 [-0.21;0.22] | *0.022* | 0.079 |
| miR-103a-3p | 88 | 0.05 [-0.10;0.20] | 88 | -0.02 [-0.10;0.12] | *0.030* | 0.091 |
| miR-106a-5p | 89 | 0.03 [-0.09;0.21] | 89 | -0.01 [-0.14;0.09] | *0.007* | *0.045* |
| miR-106b-3p | 38 | 0.01 [-0.08;0.12] | 38 | 0.01 [-0.17;0.24] | 0.904 | 0.915 |
| miR-106b-5p | 83 | 0.09 [-0.06;0.19] | 83 | -0.03 [-0.17;0.08] | *0.001* | *0.015* |
| miR-107 | 88 | 0.04 [-0.04;0.18] | 88 | -0.02 [-0.16;0.08] | *<0.001* | *0.014* |
| miR-126-3p | 89 | 0.04 [-0.09;0.16] | 89 | -0.01 [-0.11;0.07] | 0.078 | 0.186 |
| miR-126-5p | 89 | 0.05 [-0.08;0.16] | 89 | -0.04 [-0.13;0.07] | *0.018* | 0.073 |
| miR-132-3p | 76 | -0.01 [-0.15;0.17] | 76 | -0.01 [-0.18;0.15] | 0.475 | 0.601 |
| miR-133b | 59 | 0.08 [-0.18;0.31] | 59 | -0.03 [-0.24;0.17] | 0.140 | 0.241 |
| miR-139-5p | 69 | 0.07 [-0.09;0.28] | 69 | -0.05 [-0.25;0.17] | *0.009* | *0.048* |
| miR-140-3p | 82 | 0.04 [-0.11;0.15] | 82 | -0.02 [-0.10;0.10] | 0.224 | 0.329 |
| miR-140-5p | 59 | -0.01 [-0.13;0.06] | 59 | 0.01 [-0.13;0.14] | 0.110 | 0.209 |
| miR-141-3p | 33 | -0.17 [-0.33;-0.05] | 33 | -0.09 [-0.22;0.06] | 0.133 | 0.233 |
| miR-142-3p | 89 | 0.05 [-0.13;0.18] | 89 | 0.03 [-0.15;0.13] | 0.108 | 0.209 |
| miR-142-5p | 85 | -0.00 [-0.18;0.14] | 85 | 0.07 [-0.10;0.19] | 0.156 | 0.258 |
| miR-143-3p | 86 | -0.08 [-0.29;0.08] | 86 | 0.10 [-0.17;0.27] | *0.001* | *0.015* |
| miR-144-3p | 89 | 0.13 [-0.13;0.42] | 89 | -0.05 [-0.27;0.18] | *0.001* | *0.015* |
| miR-144-5p | 86 | 0.01 [-0.12;0.15] | 86 | -0.01 [-0.15;0.15] | 0.836 | 0.874 |
| miR-146a-5p | 90 | -0.01 [-0.16;0.19] | 90 | 0.02 [-0.16;0.14] | 0.562 | 0.665 |
| miR-148a-3p | 78 | -0.05 [-0.15;0.11] | 78 | 0.01 [-0.08;0.11] | 0.223 | 0.329 |
| miR-148b-3p | 85 | 0.02 [-0.07;0.13] | 85 | -0.02 [-0.10;0.07] | *0.034* | 0.101 |
| miR-151a-3p | 89 | 0.02 [-0.10;0.15] | 89 | -0.01 [-0.10;0.12] | 0.674 | 0.767 |
| miR-151a-5p | 87 | 0.06 [-0.11;0.15] | 87 | 0.01 [-0.12;0.12] | 0.180 | 0.282 |
| miR-155-5p | 63 | -0.02 [-0.17;0.24] | 63 | 0.04 [-0.19;0.13] | 0.472 | 0.601 |
| miR-15a-5p | 90 | 0.08 [-0.06;0.19] | 90 | 0.02 [-0.17;0.14] | *0.006* | *0.045* |
| miR-15b-3p | 85 | 0.05 [-0.08;0.23] | 85 | -0.03 [-0.15;0.09] | *0.004* | *0.032* |
| miR-16-2-3p | 86 | 0.07 [-0.09;0.25] | 86 | 0.00 [-0.17;0.13] | *0.012* | 0.058 |
| miR-17-5p | 88 | 0.04 [-0.13;0.33] | 88 | 0.01 [-0.12;0.29] | 0.166 | 0.270 |
| miR-181a-5p | 89 | 0.02 [-0.13;0.17] | 89 | -0.02 [-0.15;0.12] | 0.636 | 0.733 |
| miR-185-5p | 87 | 0.06 [-0.11;0.25] | 87 | -0.01 [-0.18;0.10] | *0.007* | *0.045* |
| miR-18a-5p | 88 | 0.09 [-0.06;0.17] | 88 | 0.01 [-0.14;0.10] | *<0.001* | *0.015* |
| miR-18b-5p | 87 | 0.07 [-0.10;0.23] | 87 | -0.02 [-0.17;0.11] | *0.022* | 0.079 |
| miR-191-5p | 88 | 0.03 [-0.13;0.13] | 88 | -0.00 [-0.11;0.15] | 0.726 | 0.800 |
| miR-193a-5p | 80 | -0.09 [-0.34;0.12] | 80 | 0.06 [-0.24;0.28] | 0.082 | 0.188 |
| miR-194-5p | 87 | 0.05 [-0.10;0.19] | 87 | -0.05 [-0.20;0.14] | 0.178 | 0.282 |
| miR-195-5p | 49 | 0.02 [-0.10;0.15] | 49 | 0.06 [-0.12;0.18] | 0.395 | 0.529 |
| miR-197-3p | 85 | -0.07 [-0.23;0.08] | 85 | 0.10 [-0.07;0.17] | *<0.001* | *<0.001* |
| miR-19b-3p | 89 | 0.02 [-0.13;0.19] | 89 | 0.00 [-0.11;0.09] | 0.278 | 0.383 |
| miR-200c-3p | 28 | -0.05 [-0.35;0.21] | 28 | -0.02 [-0.20;0.07] | 0.773 | 0.838 |
| miR-20a-5p | 90 | 0.03 [-0.06;0.17] | 90 | 0.02 [-0.13;0.12] | *0.021* | 0.079 |
| miR-21-5p | 87 | 0.02 [-0.06;0.13] | 87 | -0.04 [-0.15;0.05] | *0.023* | 0.079 |
| miR-210-3p | 87 | 0.03 [-0.11;0.21] | 87 | -0.01 [-0.25;0.18] | 0.055 | 0.144 |
| miR-223-3p | 86 | -0.11 [-0.27;0.06] | 86 | 0.12 [-0.18;0.24] | *<0.001* | *0.006* |
| miR-223-5p | 81 | -0.11 [-0.24;0.10] | 81 | 0.04 [-0.13;0.22] | *0.008* | 0.045 |
| miR-23a-3p | 90 | -0.02 [-0.15;0.09] | 90 | 0.02 [-0.08;0.13] | *0.047* | 0.126 |
| miR-27a-3p | 90 | -0.00 [-0.20;0.16] | 90 | 0.07 [-0.10;0.18] | *0.025* | 0.080 |
| miR-27b-3p | 86 | 0.04 [-0.12;0.14] | 86 | 0.02 [-0.09;0.13] | 0.239 | 0.335 |
| miR-28-5p | 83 | 0.05 [-0.19;0.21] | 83 | 0.01 [-0.13;0.15] | 0.937 | 0.937 |
| miR-29a-3p | 89 | -0.06 [-0.14;0.09] | 89 | 0.01 [-0.11;0.18] | *0.014* | 0.066 |
| miR-29c-3p | 83 | 0.06 [-0.10;0.16] | 83 | 0.01 [-0.16;0.13] | 0.080 | 0.186 |
| miR-301a-3p | 83 | 0.08 [-0.08;0.18] | 83 | 0.01 [-0.14;0.10] | *0.043* | 0.122 |
| miR-30b-5p | 88 | 0.02 [-0.07;0.15] | 88 | -0.03 [-0.12;0.06] | *0.007* | *0.045* |
| miR-30d-5p | 88 | -0.01 [-0.08;0.08] | 88 | -0.01 [-0.11;0.09] | 0.628 | 0.732 |
| miR-320a | 87 | 0.02 [-0.14;0.16] | 87 | -0.04 [-0.16;0.14] | 0.098 | 0.209 |
| miR-320b | 73 | 0.04 [-0.13;0.19] | 73 | -0.04 [-0.16;0.10] | 0.111 | 0.209 |
| miR-320c | 72 | -0.01 [-0.12;0.20] | 72 | -0.03 [-0.23;0.16] | 0.230 | 0.329 |
| miR-320d | 41 | 0.03 [-0.14;0.20] | 41 | -0.08 [-0.26;0.17] | 0.128 | 0.228 |
| miR-324-3p | 68 | 0.03 [-0.09;0.17] | 68 | -0.01 [-0.14;0.08] | 0.151 | 0.254 |
| miR-324-5p | 82 | 0.03 [-0.09;0.18] | 82 | -0.02 [-0.18;0.14] | 0.211 | 0.326 |
| miR-326 | 54 | -0.03 [-0.11;0.14] | 54 | 0.00 [-0.21;0.17] | 0.848 | 0.877 |
| miR-328-3p | 82 | 0.03 [-0.17;0.18] | 82 | -0.01 [-0.12;0.13] | 0.729 | 0.800 |
| miR-335-5p | 57 | 0.07 [-0.18;0.25] | 57 | -0.00 [-0.24;0.18] | 0.119 | 0.217 |
| miR-338-3p | 86 | -0.11 [-0.30;0.13] | 86 | 0.08 [-0.22;0.28] | *0.008* | *0.045* |
| miR-339-5p | 83 | 0.04 [-0.13;0.26] | 83 | -0.02 [-0.15;0.14] | 0.428 | 0.564 |
| miR-34a-5p | 80 | 0.02 [-0.30;0.16] | 80 | -0.02 [-0.24;0.26] | 0.373 | 0.507 |
| miR-362-3p | 80 | -0.00 [-0.15;0.17] | 80 | 0.03 [-0.16;0.15] | 0.905 | 0.915 |
| miR-363-3p | 89 | 0.05 [-0.09;0.21] | 89 | -0.04 [-0.14;0.11] | 0.071 | 0.174 |
| miR-365a-3p | 83 | -0.07 [-0.33;0.10] | 83 | 0.07 [-0.14;0.27] | *0.002* | *0.025* |
| miR-374a-5p | 88 | 0.03 [-0.10;0.21] | 88 | 0.05 [-0.14;0.20] | 0.785 | 0.841 |
| miR-376a-3p | 46 | 0.01 [-0.18;0.22] | 46 | -0.03 [-0.27;0.18] | 0.532 | 0.656 |
| miR-423-3p | 90 | 0.02 [-0.11;0.15] | 90 | -0.01 [-0.11;0.13] | 0.544 | 0.660 |
| miR-423-5p | 87 | 0.08 [-0.18;0.21] | 87 | 0.01 [-0.18;0.18] | 0.219 | 0.329 |
| miR-425-5p | 85 | 0.01 [-0.08;0.15] | 85 | 0.01 [-0.10;0.06] | 0.087 | 0.193 |
| miR-484 | 86 | 0.04 [-0.05;0.14] | 86 | -0.04 [-0.15;0.07] | *0.024* | 0.080 |
| miR-485-3p | 50 | 0.05 [-0.17;0.31] | 50 | -0.10 [-0.28;0.12] | *0.016* | 0.070 |
| miR-486-5p | 89 | 0.08 [-0.16;0.33] | 89 | -0.03 [-0.18;0.15] | *0.044* | 0.122 |
| miR-532-5p | 88 | 0.02 [-0.16;0.19] | 88 | 0.02 [-0.12;0.15] | 0.706 | 0.793 |
| miR-584-5p | 42 | 0.03 [-0.13;0.33] | 42 | -0.00 [-0.23;0.11] | *0.028* | 0.089 |
| miR-652-3p | 88 | 0.08 [-0.04;0.16] | 88 | -0.02 [-0.11;0.08] | *0.010* | 0.053 |
| miR-660-5p | 87 | 0.01 [-0.09;0.14] | 87 | 0.00 [-0.13;0.12] | 0.463 | 0.601 |
| miR-92a-3p | 89 | 0.04 [-0.11;0.21] | 89 | -0.02 [-0.16;0.12] | 0.102 | 0.209 |
| miR-99b-5p | 74 | 0.09 [-0.21;0.27] | 74 | -0.05 [-0.25;0.14] | 0.108 | 0.209 |
| miR-378a-3p | 56 | -0.02 [-0.13;0.11] | 56 | 0.00 [-0.14;0.16] | 0.551 | 0.660 |
