# Supplementary material for: Circulating biomarkers at diagnosis correlate with distant metastases of early luminal-like breast cancer
Source: Genes Immun. 2023 Sep 27;24(5):270–9. doi: 10.1038/s41435-023-00220-z (PMC10575765; doi:10.1038/s41435-023-00220-z)
Supplement: Supplementary file 2 — Supplementary Methods S2 [file 41435_2023_220_MOESM2_ESM.docx]

**Supplementary methods S2**

**Plasma and serum collection**

Peripheral blood was routinely sampled at first consultation in the breast clinic, before initiation of any treatment, in 5-mL BD Vacutainer® SST II Advance tubes (for serum collection) and in 4-mL BD Vacutainer® EDTA K2E tubes (for plasma collection). Blood samples were incubated at room temperature for a minimum of 20 minutes to a maximum of 60 minutes. After that, blood tubes were centrifuged at 1600xg for 15 minutes at 4°C. After centrifugation, plasma/serum was isolated and stored in aliquots at -80°C until future use. Both serum and plasma samples were used depending on availability, but for the same matched patient pair, always the same type of blood sample was used.

**Chemokine, OPN, and LIF assessments**

For the chemokine measurements, plasma samples were analyzed by cytometric bead array technology using customized multiplex chemokine panels, including fractalkine/CX3CL1, GROα/CXCL1, IP-10/CXCL10, TECK/CCL25, TARC/CCL17, IL-8/CXCL8, MCP-1/CCL2, ITAC/CXCL11, BCA-1/CXCL13, RANTES/CCL5, MIP-3b/CCL19, CTACK/CCL27, MIP-3α/CCL20, 6-Ckine/CCL21, and CXCL12/SDF-1 (Aimplex®, Biosciences Inc) and flow cytometry (FACSVerse™, BD). The assay was performed according to the manufacturer’s protocol and every sample was analyzed in duplicate. After the staining procedure, standard dilutions and samples were run on a FACSVerse flow cytometer equipped with FACSuite software (version 1.0.6, BD Biosciences). Fitting curves of the standard series and final concentrations of the samples (pg/mL) were calculated for each of the 15 analytes in FCAP Array analysis software (version 3.0, BD Biosciences).

For the plasma OPN and LIF measurements, a 2-plex bead-based immunoassay (LEGENDplex™ Custom Human Panel, BioLegend®) was performed according to the manufacturer’s protocol. After flow cytometric analysis (see above) of the standard dilutions and samples, data were analyzed with the online BioLegend LEGENDplex™ (https://www.biolegend.com/en-us/legendplex) software tool.

**Micro-RNA (miRNA) isolation**

Samples were thawed and centrifuged at 3000xg for 5 minutes at 4°C to remove debris. In order to detect hemolysis, the absorbance spectrum at 415 nm (i.e., absorbance maximum of hemoglobin) of the supernatant was measured on the Nanodrop™ 2000/2000c spectrophotometer (ThermoFisher Scientific). Hemolysis can interfere with plasma/serum miRNA profiles by contamination of cell-derived miRNAs. The qPCR quality control panel (see below) further verified the absence of hemolysis. Samples suspected of hemolysis by both methods were excluded from the experiment. Total RNA (including miRNA) was purified from non-hemolyzed samples using the miRNeasy Serum/Plasma Advanced Kit (Qiagen), following the manufacturer’s protocol with slight modifications. Concisely, to 200 µL of plasma or serum, a lysis mix was added that consists of 60 µL lysis buffer from the kit together with 1 µg of carrier M2S RNA (Roche®), and 1 µL RNA spike-in template mixture containing UniSp2, Unisp4, and Unisp5 to control for RNA extraction efficiency of low-abundance miRNAs (miRNeasy Serum/Plasma Spike-In Control, Qiagen). The following steps of miRNA isolation were carried out according to the manufacturer’s protocol. The final elution from the column was performed with 20 µL DEPC-treated RNAse-free water, and eluates were stored at -80°C until cDNA synthesis.

**cDNA synthesis and RT-qPCR-based miRNA assay**

cDNA was synthesized from 4 µL RNA extract in a 20 µL reaction volume, using the miRCURY LNA RT kit (Qiagen) according to the manufacturer’s protocol. After that, 2 µL cDNA was immediately used for quality control (QC) qPCR, using the miRCURY LNA miRNA QC PCR panel (Qiagen). The remaining 18 µL was stored at -80°C. Samples that passed the QC criteria were further analyzed in a customized PCR panel assay (96-well miRCURY LNA miRNA Custom PCR Panel, Qiagen). The panel consisted of a reduced version (1x96 well format) of the miRCURY LNA miRNA Serum/Plasma Focus PCR Panel (2x96 well format), designed for profiling of 175 human miRNAs commonly found in plasma and serum. The selection of 91 miRNAs for the reduced panel was based on a small pilot study using a subset of the samples. MiRNAs that did not give a reliable signal were removed from the panel for assessment of the remaining samples and for the final data analysis. The final list of included miRNAs in the reduced panel is shown in Supplementary Table S1. Along with the 91 selected miRNAs, the customized 96-well panel also included triplicate wells of an interplate calibrator (UniSp3) for detection of technical run-to-run differences, UniSp6 as a control for cDNA reaction efficiency, and a negative control. All PCR reactions were prepared by mixing 50-fold diluted cDNA with an equal volume of SYBR Green master mix (miRCURY LNA SYBR Green PCR Kit, Qiagen). The final reaction volume was 10 µL per well. PCR plates were run on the LightCycler®480 (LC480, Roche) instrument applying the following thermal cycling protocol: activation step (10 min at 95°C); 45 amplification cycles (10 s at 95°C, 1 min at 60°C, ramp rate 1,6°C/s); melting curve (5 s at 95°C, 1 min at 40°C); cool down (10 s at 40°C). After RT-qPCR, Cp values were determined by the LC480 software using the second derivative method and were exported for further analysis in the qBase+ software (version 3.1, Biogazelle). Global mean normalization was applied and results were calculated as logarithmically (log10 base) transformed calibrated normalized relative quantities (CNRQ values), which were used for further statistical analysis.

**Serum functional immunodynamic status (sFIS) assay**

Baseline serum samples were analyzed with the sFIS assay. THP1-DualTM cells (InvivoGen) featuring simultaneously a secreted embryonic alkaline phosphatase (SEAP) reporter gene and a secreted luciferase reporter gene driven by their specific promoters, resistant to the selectable markers zeocin and blasticidin, were used to study the NFκB pathway and the interferon regulatory factors (IRF) pathway by monitoring, respectively, the activity of SEAP and Lucia luciferase. The THP-1 dual cells were cultured at 37°C under 5% CO_2_ pressure in RPMI-1640 medium containing 10% heat-inactivated fetal bovine serum (FBS), 100 U/mL penicillin, 100 µg/mL streptomycin, 100 µg/mL normocin, 2 mM L-glutamine, 25 mM 4-(2-hydroxyethyl)-1-piperazineethanesulfonic acid (HEPES). For every other passage, the cells were cultured with 10 µg/mL blasticidin and 100 µg/mL zeocin to select the dual reporter expressing cells. For analysis of human serum samples, THP1-DualTM cells (InvivoGen) were seeded in 100 µL medium a density of 30 000 cells/well in a 96-well plate. After 24 h, THP1 cells were treated with 1000 ng/mL lipopolysaccharide as a positive control, 100 µL of normal human serum (pooled from several normal human individuals, Sigma-Aldrich) as a baseline for the assay, or with patient serum of the trial. Luciferase activity in the media was checked after adding 50 µL of Quanti-Luc (Invivogen) to 100 µL of (separately recovered) THP1 media by examining the bioluminescence for 100 ms immediately after adding Quanti-Luc. SEAP activity was assessed by adding 100 µL of Quanti-Blue (Invivogen) to 100 µL of (separately recovered) THP1 media and reading the absorbance at an optical density of 655 nm, 4–8 h after Quanti-Blue addition. The measurement of both luciferase (bioluminescence) and SEAP (colorimetric) activity was performed with a microplate reader (Biotek) (1, 2).

**References**

1. Sprooten J, Coosemans A, Garg AD. A first-in-class, non-invasive, immunodynamic biomarker approach for precision immuno-oncology. Oncoimmunology. 2022;11(1):2024692.

2. Sprooten J, Vankerckhoven A, Vanmeerbeek I, Borras DM, Berckmans Y, Wouters R, et al. Peripherally-driven myeloid NFkB and IFN/ISG responses predict malignancy risk, survival, and immunotherapy regime in ovarian cancer. J Immunother Cancer. 2021;9(11).
