# Supplementary material for: Circulating biomarkers at diagnosis correlate with distant metastases of early luminal-like breast cancer
Source: Genes Immun. 2023 Sep 27;24(5):270–9. doi: 10.1038/s41435-023-00220-z (PMC10575765; doi:10.1038/s41435-023-00220-z)
Supplement: Supplementary file 3 — Supplementary Table S3: Baseline plasma circulating chemokine differences between the early-relapsing (META) versus non-relapsing (NON-META) patients [file 41435_2023_220_MOESM3_ESM.docx]

**Supplementary Table S3**

*Supplementary Table S3: Baseline plasma circulating chemokine differences between the early-relapsing (META) versus non-relapsing (NON-META) patients. The number of patients included in this analysis, median, interquartile range (IQR), raw P-value, and FDR-corrected p-value are reported. The median and IQR measurements are reported in pg/mL. The P-values were calculated using a paired Wilcoxon signed-rank test. The P-values that were statistically significant are reported in italics. FDR: false discovery rate.*

|  | **NON-META** | | **META** | | ***P*-values** | |
| --- | --- | --- | --- | --- | --- | --- |
| **Biomarker** | **N** | **Median [IQR]** | **N** | **Median [IQR]** | **Raw** | **FDR** |
| Fractalkine | 102 | 54.69 [45.44;71.15] | 102 | 52.24 [39.73;73.20] | 0.156 | 0.234 |
| GROalpha | 102 | 30.93 [10.74;126.71] | 102 | 36.65 [9.08;102.54] | 0.107 | 0.201 |
| IP-10 | 102 | 14.55 [9.58;143.57] | 102 | 16.31 [10.06;171.27] | *0.024* | 0.091 |
| TECK | 102 | 7.37 [1.92;59.84] | 102 | 9.83 [2.08;63.73] | 0.067 | 0.200 |
| TARC | 102 | 53.09 [36.74;77.70] | 102 | 46.51 [35.31;61.34] | *0.011* | 0.088 |
| IL-8 | 102 | 15.13 [11.48;19.90] | 102 | 13.12 [9.79;17.83] | 0.148 | 0.234 |
| MCP-1 | 102 | 31.43 [23.07;42.84] | 102 | 31.43 [22.22;39.72] | 0.462 | 0.623 |
| ITAC | 102 | 11.43 [5.96;24.00] | 102 | 13.45 [5.96;23.63] | 0.922 | 0.936 |
| BCA-1 | 102 | 17.52 [7.48;39.80] | 102 | 20.85 [7.25;41.78] | 0.754 | 0.871 |
| RANTES | 102 | 2335.3 [2060.2;2847.9] | 102 | 2175.5 [1896.6;2624.5] | *0.012* | 0.088 |
| MIP-3beta | 102 | 0.00 [0.00;119.56] | 102 | 5.51 [0.00;117.68] | 0.936 | 0.936 |
| CTACK | 102 | 683.32 [329.47;1017.9] | 102 | 639.43 [298.27;977.96] | *0.020* | 0.091 |
| MIP-3alpha | 102 | 96.62 [22.38;162.12] | 102 | 97.18 [19.22;149.72] | 0.102 | 0.201 |
| 6-Ckine | 102 | 217.17 [154.22;281.55] | 102 | 227.30 [130.25;287.85] | 0.649 | 0.811 |
| SDF-1 | 102 | 18.11 [0.00;98.47] | 102 | 20.51 [4.00;112.69] | 0.107 | 0.201 |
