# Supplementary material for: Circulating biomarkers at diagnosis correlate with distant metastases of early luminal-like breast cancer
Source: Genes Immun. 2023 Sep 27;24(5):270–9. doi: 10.1038/s41435-023-00220-z (PMC10575765; doi:10.1038/s41435-023-00220-z)
Supplement: Supplementary file 4 — Supplementary Table S4: Pathway ranking summary of the pathway analysis from the REACTOME software. [file 41435_2023_220_MOESM4_ESM.docx]

**Supplementary Table S4**

*Supplementary Table S4: Pathway ranking summary of the pathway analysis from the REACTOME software. The represented pathways are analyzed from the miRNAs highly expressed in non-metastasizing early luminal breast cancer patients. The score represents the alignment of multiple miRNAs involved in pathways in Homo Sapiens. The higher the score, the better the alignment of the multiple miRNAs with that particular pathway.*

| **TERM** | **TERM_ID** | **URL** | **TOTAL_GENES_OF_THE_TERM** | **UNION_TARGETS_IN_THE_TERM** | **MIRS_IN_THE_TERM** | **SCORE** |
| --- | --- | --- | --- | --- | --- | --- |
| SIGNAL_AMPLIFICATION | REACT_20524 | http://www.reactome.org/cgi-bin/eventbrowser_st_id?ST_ID=REACT_20524 | 31 | 11 | 10 | 0,51 |
| RNA_POLYMERASE_II_PRE-TRANSCRIPTION_EVENTS | REACT_22107 | http://www.reactome.org/cgi-bin/eventbrowser_st_id?ST_ID=REACT_22107 | 58 | 9 | 10 | 0,54 |
| RNA_POLYMERASE_II_TRANSCRIPTION | REACT_1366 | http://www.reactome.org/cgi-bin/eventbrowser_st_id?ST_ID=REACT_1366 | 101 | 16 | 10 | 0,55 |
| INTERLEUKIN_RECEPTOR_SHC_SIGNALING | REACT_23891 | http://www.reactome.org/cgi-bin/eventbrowser_st_id?ST_ID=REACT_23891 | 28 | 12 | 10 | 0,57 |
| P53-DEPENDENT_G1_DNA_DAMAGE_RESPONSE | REACT_1625 | http://www.reactome.org/cgi-bin/eventbrowser_st_id?ST_ID=REACT_1625 | 57 | 11 | 10 | 0,57 |
| P53-DEPENDENT_G1_S_DNA_DAMAGE_CHECKPOINT | REACT_85 | http://www.reactome.org/cgi-bin/eventbrowser_st_id?ST_ID=REACT_85 | 57 | 11 | 10 | 0,57 |
| ADHERENS_JUNCTIONS_INTERACTIONS | REACT_19195 | http://www.reactome.org/cgi-bin/eventbrowser_st_id?ST_ID=REACT_19195 | 29 | 12 | 10 | 0,59 |
| S_PHASE | REACT_899 | http://www.reactome.org/cgi-bin/eventbrowser_st_id?ST_ID=REACT_899 | 112 | 17 | 10 | 0,61 |
| MUSCLE_CONTRACTION | REACT_17044 | http://www.reactome.org/cgi-bin/eventbrowser_st_id?ST_ID=REACT_17044 | 49 | 10 | 10 | 0,61 |
| **INTERFERON_GAMMA_SIGNALING** | REACT_25078 | http://www.reactome.org/cgi-bin/eventbrowser_st_id?ST_ID=REACT_25078 | 73 | 17 | 11 | 0,62 |
| TRANSCRIPTION_OF_THE_HIV_GENOME | REACT_6233 | http://www.reactome.org/cgi-bin/eventbrowser_st_id?ST_ID=REACT_6233 | 61 | 10 | 10 | 0,62 |
| SHC-MEDIATED_CASCADE | REACT_21374 | http://www.reactome.org/cgi-bin/eventbrowser_st_id?ST_ID=REACT_21374 | 29 | 11 | 10 | 0,63 |
| REGULATION_OF_GENE_EXPRESSION_IN_BETA_CELLS | REACT_13819 | http://www.reactome.org/cgi-bin/eventbrowser_st_id?ST_ID=REACT_13819 | 102 | 6 | 10 | 0,64 |
| GLUTAMATE_BINDING_ACTIVATION_OF_AMPA_RECEPTORS_AND_SYNAPTIC_PLASTICITY | REACT_18347 | http://www.reactome.org/cgi-bin/eventbrowser_st_id?ST_ID=REACT_18347 | 30 | 10 | 10 | 0,64 |
| TRAFFICKING_OF_AMPA_RECEPTORS | REACT_18307 | http://www.reactome.org/cgi-bin/eventbrowser_st_id?ST_ID=REACT_18307 | 30 | 10 | 10 | 0,64 |
| CELL_CYCLE_CHECKPOINTS | REACT_1538 | http://www.reactome.org/cgi-bin/eventbrowser_st_id?ST_ID=REACT_1538 | 117 | 21 | 10 | 0,65 |
| G1_S_DNA_DAMAGE_CHECKPOINTS | REACT_2254 | http://www.reactome.org/cgi-bin/eventbrowser_st_id?ST_ID=REACT_2254 | 60 | 13 | 10 | 0,66 |
| NOD1_2_SIGNALING_PATHWAY | REACT_75776 | http://www.reactome.org/cgi-bin/eventbrowser_st_id?ST_ID=REACT_75776 | 31 | 12 | 10 | 0,66 |
| MEIOSIS | REACT_111183 | http://www.reactome.org/cgi-bin/eventbrowser_st_id?ST_ID=REACT_111183 | 85 | 15 | 10 | 0,67 |
| APOPTOTIC_EXECUTION_PHASE | REACT_995 | http://www.reactome.org/cgi-bin/eventbrowser_st_id?ST_ID=REACT_995 | 52 | 17 | 10 | 0,67 |
| TRANSCRIPTION | REACT_1788 | http://www.reactome.org/cgi-bin/eventbrowser_st_id?ST_ID=REACT_1788 | 177 | 29 | 10 | 0,67 |
| APOPTOTIC_CLEAVAGE_OF_CELLULAR_PROTEINS | REACT_107 | http://www.reactome.org/cgi-bin/eventbrowser_st_id?ST_ID=REACT_107 | 38 | 14 | 10 | 0,68 |
| CELL_JUNCTION_ORGANIZATION | REACT_20676 | http://www.reactome.org/cgi-bin/eventbrowser_st_id?ST_ID=REACT_20676 | 84 | 26 | 10 | 0,68 |
| **INTERFERON_SIGNALING** | REACT_25229 | http://www.reactome.org/cgi-bin/eventbrowser_st_id?ST_ID=REACT_25229 | 110 | 21 | 11 | 0,68 |
| G_ALPHA_(Q)_SIGNALLING_EVENTS | REACT_18283 | http://www.reactome.org/cgi-bin/eventbrowser_st_id?ST_ID=REACT_18283 | 186 | 41 | 10 | 0,68 |
| TRANSPORT_OF_GLUCOSE_AND_OTHER_SUGARS_BILE_SALTS_AND_ORGANIC_ACIDS_METAL_IONS_AND_AMINE_COMPOUNDS | REACT_19305 | http://www.reactome.org/cgi-bin/eventbrowser_st_id?ST_ID=REACT_19305 | 96 | 28 | 10 | 0,69 |
| CENTROSOME_MATURATION | REACT_15479 | http://www.reactome.org/cgi-bin/eventbrowser_st_id?ST_ID=REACT_15479 | 72 | 19 | 11 | 0,70 |
| RECRUITMENT_OF_MITOTIC_CENTROSOME_PROTEINS_AND_COMPLEXES | REACT_15296 | http://www.reactome.org/cgi-bin/eventbrowser_st_id?ST_ID=REACT_15296 | 72 | 19 | 11 | 0,70 |
| NEGATIVE_REGULATORS_OF_RIG-I_MDA5_SIGNALING | REACT_25271 | http://www.reactome.org/cgi-bin/eventbrowser_st_id?ST_ID=REACT_25271 | 33 | 11 | 10 | 0,70 |
| REGULATION_OF_BETA-CELL_DEVELOPMENT | REACT_13698 | http://www.reactome.org/cgi-bin/eventbrowser_st_id?ST_ID=REACT_13698 | 114 | 9 | 10 | 0,70 |
| BIOLOGICAL_OXIDATIONS | REACT_13433 | http://www.reactome.org/cgi-bin/eventbrowser_st_id?ST_ID=REACT_13433 | 139 | 16 | 10 | 0,71 |
| GLUCAGON_SIGNALING_IN_METABOLIC_REGULATION | REACT_1665 | http://www.reactome.org/cgi-bin/eventbrowser_st_id?ST_ID=REACT_1665 | 33 | 12 | 10 | 0,71 |
| INTERLEUKIN-2_SIGNALING | REACT_27283 | http://www.reactome.org/cgi-bin/eventbrowser_st_id?ST_ID=REACT_27283 | 42 | 19 | 10 | 0,72 |
| LIPID_DIGESTION_MOBILIZATION_AND_TRANSPORT | REACT_602 | http://www.reactome.org/cgi-bin/eventbrowser_st_id?ST_ID=REACT_602 | 48 | 13 | 10 | 0,72 |
| AQUAPORIN-MEDIATED_TRANSPORT | REACT_23887 | http://www.reactome.org/cgi-bin/eventbrowser_st_id?ST_ID=REACT_23887 | 47 | 16 | 11 | 0,72 |
| G1_S_TRANSITION | REACT_1783 | http://www.reactome.org/cgi-bin/eventbrowser_st_id?ST_ID=REACT_1783 | 109 | 22 | 11 | 0,72 |
| CELL-CELL_JUNCTION_ORGANIZATION | REACT_19331 | http://www.reactome.org/cgi-bin/eventbrowser_st_id?ST_ID=REACT_19331 | 59 | 19 | 10 | 0,75 |
| E2F_MEDIATED_REGULATION_OF_DNA_REPLICATION | REACT_471 | http://www.reactome.org/cgi-bin/eventbrowser_st_id?ST_ID=REACT_471 | 33 | 11 | 10 | 0,75 |
| HOST_INTERACTIONS_OF_HIV_FACTORS | REACT_6288 | http://www.reactome.org/cgi-bin/eventbrowser_st_id?ST_ID=REACT_6288 | 135 | 34 | 10 | 0,76 |
| LOSS_OF_NLP_FROM_MITOTIC_CENTROSOMES | REACT_15364 | http://www.reactome.org/cgi-bin/eventbrowser_st_id?ST_ID=REACT_15364 | 62 | 18 | 11 | 0,76 |
| LOSS_OF_PROTEINS_REQUIRED_FOR_INTERPHASE_MICROTUBULE_ORGANIZATION??FROM_THE_CENTROSOME | REACT_15451 | http://www.reactome.org/cgi-bin/eventbrowser_st_id?ST_ID=REACT_15451 | 62 | 18 | 11 | 0,76 |
| METABOLISM_OF_AMINO_ACIDS_AND_DERIVATIVES | REACT_13 | http://www.reactome.org/cgi-bin/eventbrowser_st_id?ST_ID=REACT_13 | 174 | 23 | 10 | 0,76 |
| REGULATION_OF_WATER_BALANCE_BY_RENAL_AQUAPORINS | REACT_24023 | http://www.reactome.org/cgi-bin/eventbrowser_st_id?ST_ID=REACT_24023 | 40 | 16 | 11 | 0,77 |
| MITOTIC_PROMETAPHASE | REACT_682 | http://www.reactome.org/cgi-bin/eventbrowser_st_id?ST_ID=REACT_682 | 92 | 27 | 11 | 0,77 |
| MITOTIC_M-M_G1_PHASES | REACT_21300 | http://www.reactome.org/cgi-bin/eventbrowser_st_id?ST_ID=REACT_21300 | 178 | 39 | 11 | 0,78 |
| PLATELET_AGGREGATION_(PLUG_FORMATION) | REACT_278 | http://www.reactome.org/cgi-bin/eventbrowser_st_id?ST_ID=REACT_278 | 37 | 12 | 10 | 0,78 |
| G_ALPHA_(I)_SIGNALLING_EVENTS | REACT_19231 | http://www.reactome.org/cgi-bin/eventbrowser_st_id?ST_ID=REACT_19231 | 200 | 42 | 11 | 0,78 |
| TRANSCRIPTIONAL_REGULATION_OF_WHITE_ADIPOCYTE_DIFFERENTIATION | REACT_27161 | http://www.reactome.org/cgi-bin/eventbrowser_st_id?ST_ID=REACT_27161 | 69 | 27 | 11 | 0,79 |
| FORMATION_AND_MATURATION_OF_MRNA_TRANSCRIPT | REACT_2039 | http://www.reactome.org/cgi-bin/eventbrowser_st_id?ST_ID=REACT_2039 | 185 | 40 | 10 | 0,79 |
| SIGNALLING_TO_ERKS | REACT_12058 | http://www.reactome.org/cgi-bin/eventbrowser_st_id?ST_ID=REACT_12058 | 35 | 15 | 10 | 0,80 |
| ACTIVATION_OF_GABAB_RECEPTORS | REACT_25330 | http://www.reactome.org/cgi-bin/eventbrowser_st_id?ST_ID=REACT_25330 | 38 | 16 | 11 | 0,80 |
| GABA_B_RECEPTOR_ACTIVATION | REACT_25031 | http://www.reactome.org/cgi-bin/eventbrowser_st_id?ST_ID=REACT_25031 | 38 | 16 | 11 | 0,80 |
| G2_M_TRANSITION | REACT_2203 | http://www.reactome.org/cgi-bin/eventbrowser_st_id?ST_ID=REACT_2203 | 84 | 24 | 11 | 0,81 |
| M_PHASE | REACT_910 | http://www.reactome.org/cgi-bin/eventbrowser_st_id?ST_ID=REACT_910 | 96 | 28 | 11 | 0,81 |
| LATE_PHASE_OF_HIV_LIFE_CYCLE | REACT_6361 | http://www.reactome.org/cgi-bin/eventbrowser_st_id?ST_ID=REACT_6361 | 94 | 22 | 10 | 0,81 |
| G_ALPHA_(Z)_SIGNALLING_EVENTS | REACT_19333 | http://www.reactome.org/cgi-bin/eventbrowser_st_id?ST_ID=REACT_19333 | 45 | 18 | 11 | 0,81 |
| DNA_REPLICATION | REACT_383 | http://www.reactome.org/cgi-bin/eventbrowser_st_id?ST_ID=REACT_383 | 200 | 42 | 11 | 0,81 |
| PIP3_ACTIVATES_AKT_SIGNALING | REACT_75829 | http://www.reactome.org/cgi-bin/eventbrowser_st_id?ST_ID=REACT_75829 | 28 | 10 | 10 | 0,82 |
| NUCLEOTIDE-BINDING_DOMAIN_LEUCINE_RICH_REPEAT_CONTAINING_RECEPTOR_(NLR)_SIGNALING_PATHWAYS | REACT_75913 | http://www.reactome.org/cgi-bin/eventbrowser_st_id?ST_ID=REACT_75913 | 51 | 16 | 11 | 0,83 |
| SEMA4D_IN_SEMAPHORIN_SIGNALING | REACT_19259 | http://www.reactome.org/cgi-bin/eventbrowser_st_id?ST_ID=REACT_19259 | 29 | 14 | 11 | 0,83 |
| HIV_LIFE_CYCLE | REACT_6256 | http://www.reactome.org/cgi-bin/eventbrowser_st_id?ST_ID=REACT_6256 | 113 | 24 | 10 | 0,83 |
| **RIG-I_MDA5_MEDIATED_INDUCTION_OF_IFN-ALPHA_BETA_PATHWAYS** | REACT_25359 | http://www.reactome.org/cgi-bin/eventbrowser_st_id?ST_ID=REACT_25359 | 76 | 20 | 11 | 0,83 |
| DEADENYLATION-DEPENDENT_MRNA_DECAY | REACT_20639 | http://www.reactome.org/cgi-bin/eventbrowser_st_id?ST_ID=REACT_20639 | 46 | 13 | 10 | 0,83 |
| EGFR_INTERACTS_WITH_PHOSPHOLIPASE_C-GAMMA | REACT_12478 | http://www.reactome.org/cgi-bin/eventbrowser_st_id?ST_ID=REACT_12478 | 33 | 14 | 11 | 0,84 |
| NETRIN-1_SIGNALING | REACT_22237 | http://www.reactome.org/cgi-bin/eventbrowser_st_id?ST_ID=REACT_22237 | 42 | 15 | 10 | 0,84 |
| NCAM1_INTERACTIONS | REACT_18312 | http://www.reactome.org/cgi-bin/eventbrowser_st_id?ST_ID=REACT_18312 | 44 | 20 | 10 | 0,84 |
| INTEGRIN_ALPHAIIB_BETA3_SIGNALING | REACT_15523 | http://www.reactome.org/cgi-bin/eventbrowser_st_id?ST_ID=REACT_15523 | 27 | 10 | 10 | 0,84 |
| CLASS_B_2_(SECRETIN_FAMILY_RECEPTORS) | REACT_18372 | http://www.reactome.org/cgi-bin/eventbrowser_st_id?ST_ID=REACT_18372 | 90 | 29 | 11 | 0,85 |
| GLUCOSE_METABOLISM | REACT_723 | http://www.reactome.org/cgi-bin/eventbrowser_st_id?ST_ID=REACT_723 | 62 | 19 | 10 | 0,85 |
| REGULATION_OF_INSULIN_SECRETION_BY_GLUCAGON-LIKE_PEPTIDE-1 | REACT_18274 | http://www.reactome.org/cgi-bin/eventbrowser_st_id?ST_ID=REACT_18274 | 43 | 16 | 11 | 0,86 |
| DAG_AND_IP3_SIGNALING | REACT_111064 | http://www.reactome.org/cgi-bin/eventbrowser_st_id?ST_ID=REACT_111064 | 31 | 14 | 11 | 0,86 |
| PLATELET_DEGRANULATION | REACT_318 | http://www.reactome.org/cgi-bin/eventbrowser_st_id?ST_ID=REACT_318 | 78 | 24 | 11 | 0,86 |
| INSULIN_SYNTHESIS_AND_PROCESSING | REACT_15550 | http://www.reactome.org/cgi-bin/eventbrowser_st_id?ST_ID=REACT_15550 | 135 | 22 | 11 | 0,87 |
| CDO_IN_MYOGENESIS | REACT_21402 | http://www.reactome.org/cgi-bin/eventbrowser_st_id?ST_ID=REACT_21402 | 29 | 12 | 10 | 0,88 |
| MYOGENESIS | REACT_21303 | http://www.reactome.org/cgi-bin/eventbrowser_st_id?ST_ID=REACT_21303 | 29 | 12 | 10 | 0,88 |
| RESPONSE_TO_ELEVATED_PLATELET_CYTOSOLIC_CA2+ | REACT_1280 | http://www.reactome.org/cgi-bin/eventbrowser_st_id?ST_ID=REACT_1280 | 83 | 26 | 11 | 0,89 |
| CLASS_A_1_(RHODOPSIN-LIKE_RECEPTORS) | REACT_14828 | http://www.reactome.org/cgi-bin/eventbrowser_st_id?ST_ID=REACT_14828 | 305 | 48 | 10 | 0,89 |
| CELL_SURFACE_INTERACTIONS_AT_THE_VASCULAR_WALL | REACT_12051 | http://www.reactome.org/cgi-bin/eventbrowser_st_id?ST_ID=REACT_12051 | 94 | 29 | 11 | 0,90 |
| G_ALPHA_(S)_SIGNALLING_EVENTS | REACT_19327 | http://www.reactome.org/cgi-bin/eventbrowser_st_id?ST_ID=REACT_19327 | 125 | 32 | 11 | 0,90 |
| GPCR_LIGAND_BINDING | REACT_21340 | http://www.reactome.org/cgi-bin/eventbrowser_st_id?ST_ID=REACT_21340 | 410 | 84 | 11 | 0,90 |
| PEPTIDE_LIGAND-BINDING_RECEPTORS | REACT_14819 | http://www.reactome.org/cgi-bin/eventbrowser_st_id?ST_ID=REACT_14819 | 186 | 29 | 10 | 0,91 |
| METABOLISM_OF_CARBOHYDRATES | REACT_474 | http://www.reactome.org/cgi-bin/eventbrowser_st_id?ST_ID=REACT_474 | 126 | 33 | 11 | 0,92 |
| GPVI-MEDIATED_ACTIVATION_CASCADE | REACT_1695 | http://www.reactome.org/cgi-bin/eventbrowser_st_id?ST_ID=REACT_1695 | 33 | 15 | 10 | 0,92 |
| POTASSIUM_CHANNELS | REACT_75908 | http://www.reactome.org/cgi-bin/eventbrowser_st_id?ST_ID=REACT_75908 | 99 | 32 | 12 | 0,92 |
| DIABETES_PATHWAYS | REACT_15380 | http://www.reactome.org/cgi-bin/eventbrowser_st_id?ST_ID=REACT_15380 | 229 | 45 | 12 | 0,93 |
| METABOLISM_OF_PROTEINS | REACT_17015 | http://www.reactome.org/cgi-bin/eventbrowser_st_id?ST_ID=REACT_17015 | 296 | 56 | 12 | 0,94 |
| GENERIC_TRANSCRIPTION_PATHWAY | REACT_12627 | http://www.reactome.org/cgi-bin/eventbrowser_st_id?ST_ID=REACT_12627 | 244 | 57 | 11 | 0,96 |
| NEUROTRANSMITTER_RELEASE_CYCLE | REACT_13723 | http://www.reactome.org/cgi-bin/eventbrowser_st_id?ST_ID=REACT_13723 | 36 | 18 | 10 | 0,96 |
| MITOTIC_G2-G2_M_PHASES | REACT_21391 | http://www.reactome.org/cgi-bin/eventbrowser_st_id?ST_ID=REACT_21391 | 87 | 27 | 11 | 0,97 |
| APOPTOSIS | REACT_578 | http://www.reactome.org/cgi-bin/eventbrowser_st_id?ST_ID=REACT_578 | 148 | 45 | 11 | 0,98 |
| INNATE_IMMUNE_SYSTEM | REACT_6802 | http://www.reactome.org/cgi-bin/eventbrowser_st_id?ST_ID=REACT_6802 | 262 | 59 | 11 | 0,98 |
| G-PROTEIN_MEDIATED_EVENTS | REACT_15526 | http://www.reactome.org/cgi-bin/eventbrowser_st_id?ST_ID=REACT_15526 | 44 | 19 | 11 | 1,00 |
| METABOLISM_OF_MRNA | REACT_20605 | http://www.reactome.org/cgi-bin/eventbrowser_st_id?ST_ID=REACT_20605 | 218 | 38 | 11 | 1,00 |
| METABOLISM_OF_RNA | REACT_21257 | http://www.reactome.org/cgi-bin/eventbrowser_st_id?ST_ID=REACT_21257 | 264 | 50 | 11 | 1,00 |
| FATTY_ACID_TRIACYLGLYCEROL_AND_KETONE_BODY_METABOLISM | REACT_22279 | http://www.reactome.org/cgi-bin/eventbrowser_st_id?ST_ID=REACT_22279 | 112 | 35 | 11 | 1,00 |
| FRS2-MEDIATED_CASCADE | REACT_21247 | http://www.reactome.org/cgi-bin/eventbrowser_st_id?ST_ID=REACT_21247 | 38 | 16 | 11 | 1,00 |
| PLC_BETA_MEDIATED_EVENTS | REACT_15426 | http://www.reactome.org/cgi-bin/eventbrowser_st_id?ST_ID=REACT_15426 | 43 | 18 | 11 | 1,01 |
| TRANSPORT_TO_THE_GOLGI_AND_SUBSEQUENT_MODIFICATION | REACT_25046 | http://www.reactome.org/cgi-bin/eventbrowser_st_id?ST_ID=REACT_25046 | 36 | 16 | 12 | 1,01 |
| ASPARAGINE_N-LINKED_GLYCOSYLATION | REACT_22426 | http://www.reactome.org/cgi-bin/eventbrowser_st_id?ST_ID=REACT_22426 | 85 | 26 | 12 | 1,02 |
| GABA_RECEPTOR_ACTIVATION | REACT_25199 | http://www.reactome.org/cgi-bin/eventbrowser_st_id?ST_ID=REACT_25199 | 53 | 23 | 12 | 1,02 |
| METABOLISM_OF_LIPIDS_AND_LIPOPROTEINS | REACT_22258 | http://www.reactome.org/cgi-bin/eventbrowser_st_id?ST_ID=REACT_22258 | 292 | 77 | 12 | 1,04 |
| PHOSPHOLIPASE_C-MEDIATED_CASCADE | REACT_21310 | http://www.reactome.org/cgi-bin/eventbrowser_st_id?ST_ID=REACT_21310 | 54 | 22 | 11 | 1,04 |
| HIV_INFECTION | REACT_6185 | http://www.reactome.org/cgi-bin/eventbrowser_st_id?ST_ID=REACT_6185 | 200 | 45 | 11 | 1,06 |
| PLC-GAMMA1_SIGNALLING | REACT_12079 | http://www.reactome.org/cgi-bin/eventbrowser_st_id?ST_ID=REACT_12079 | 34 | 16 | 11 | 1,06 |
| CELL-CELL_COMMUNICATION | REACT_111155 | http://www.reactome.org/cgi-bin/eventbrowser_st_id?ST_ID=REACT_111155 | 129 | 42 | 11 | 1,08 |
| EGFR_DOWNREGULATION | REACT_12484 | http://www.reactome.org/cgi-bin/eventbrowser_st_id?ST_ID=REACT_12484 | 27 | 15 | 11 | 1,09 |
| POST-TRANSLATIONAL_PROTEIN_MODIFICATION | REACT_22161 | http://www.reactome.org/cgi-bin/eventbrowser_st_id?ST_ID=REACT_22161 | 123 | 33 | 12 | 1,10 |
| MTOR_SIGNALLING | REACT_6838 | http://www.reactome.org/cgi-bin/eventbrowser_st_id?ST_ID=REACT_6838 | 27 | 13 | 10 | 1,12 |
| GAB1_SIGNALOSOME | REACT_12578 | http://www.reactome.org/cgi-bin/eventbrowser_st_id?ST_ID=REACT_12578 | 39 | 15 | 10 | 1,14 |
| REGULATION_OF_INSULIN_SECRETION | REACT_18325 | http://www.reactome.org/cgi-bin/eventbrowser_st_id?ST_ID=REACT_18325 | 98 | 36 | 12 | 1,14 |
| SLC-MEDIATED_TRANSMEMBRANE_TRANSPORT | REACT_19118 | http://www.reactome.org/cgi-bin/eventbrowser_st_id?ST_ID=REACT_19118 | 250 | 73 | 11 | 1,15 |
| INTEGRIN_CELL_SURFACE_INTERACTIONS | REACT_13552 | http://www.reactome.org/cgi-bin/eventbrowser_st_id?ST_ID=REACT_13552 | 85 | 28 | 11 | 1,16 |
| TRANSPORT_OF_INORGANIC_CATIONS_ANIONS_AND_AMINO_ACIDS_OLIGOPEPTIDES | REACT_19397 | http://www.reactome.org/cgi-bin/eventbrowser_st_id?ST_ID=REACT_19397 | 94 | 32 | 11 | 1,17 |
| INTERLEUKIN-3_5_AND_GM-CSF_SIGNALING | REACT_23837 | http://www.reactome.org/cgi-bin/eventbrowser_st_id?ST_ID=REACT_23837 | 45 | 20 | 11 | 1,17 |
| ION_CHANNEL_TRANSPORT | REACT_25300 | http://www.reactome.org/cgi-bin/eventbrowser_st_id?ST_ID=REACT_25300 | 61 | 24 | 12 | 1,18 |
| KINESINS | REACT_25201 | http://www.reactome.org/cgi-bin/eventbrowser_st_id?ST_ID=REACT_25201 | 27 | 11 | 10 | 1,20 |
| ACTIVATION_OF_NMDA_RECEPTOR_UPON_GLUTAMATE_BINDING_AND_POSTSYNAPTIC_EVENTS | REACT_20563 | http://www.reactome.org/cgi-bin/eventbrowser_st_id?ST_ID=REACT_20563 | 37 | 18 | 10 | 1,20 |
| NCAM_SIGNALING_FOR_NEURITE_OUT-GROWTH | REACT_18334 | http://www.reactome.org/cgi-bin/eventbrowser_st_id?ST_ID=REACT_18334 | 70 | 34 | 11 | 1,21 |
| PI3K_AKT_ACTIVATION | REACT_12464 | http://www.reactome.org/cgi-bin/eventbrowser_st_id?ST_ID=REACT_12464 | 37 | 16 | 10 | 1,23 |
| TOLL_LIKE_RECEPTOR_4_(TLR4)_CASCADE | REACT_6894 | http://www.reactome.org/cgi-bin/eventbrowser_st_id?ST_ID=REACT_6894 | 96 | 34 | 11 | 1,24 |
| MYD88-INDEPENDENT_CASCADE_INITIATED_ON_PLASMA_MEMBRANE | REACT_6809 | http://www.reactome.org/cgi-bin/eventbrowser_st_id?ST_ID=REACT_6809 | 75 | 30 | 11 | 1,24 |
| POST_NMDA_RECEPTOR_ACTIVATION_EVENTS | REACT_20593 | http://www.reactome.org/cgi-bin/eventbrowser_st_id?ST_ID=REACT_20593 | 33 | 17 | 10 | 1,25 |
| TOLL_LIKE_RECEPTOR_3_(TLR3)_CASCADE | REACT_6783 | http://www.reactome.org/cgi-bin/eventbrowser_st_id?ST_ID=REACT_6783 | 74 | 30 | 11 | 1,26 |
| TRIF_MEDIATED_TLR3_SIGNALING | REACT_111135 | http://www.reactome.org/cgi-bin/eventbrowser_st_id?ST_ID=REACT_111135 | 74 | 30 | 11 | 1,26 |
| NEGATIVE_REGULATION_OF_FGFR_SIGNALING | REACT_111184 | http://www.reactome.org/cgi-bin/eventbrowser_st_id?ST_ID=REACT_111184 | 40 | 18 | 11 | 1,26 |
| P75_NTR_RECEPTOR-MEDIATED_SIGNALLING | REACT_13776 | http://www.reactome.org/cgi-bin/eventbrowser_st_id?ST_ID=REACT_13776 | 86 | 35 | 11 | 1,27 |
| SEMAPHORIN_INTERACTIONS | REACT_19271 | http://www.reactome.org/cgi-bin/eventbrowser_st_id?ST_ID=REACT_19271 | 66 | 29 | 11 | 1,27 |
| TOLL_RECEPTOR_CASCADES | REACT_6966 | http://www.reactome.org/cgi-bin/eventbrowser_st_id?ST_ID=REACT_6966 | 108 | 36 | 11 | 1,27 |
| NRAGE_SIGNALS_DEATH_THROUGH_JNK | REACT_13638 | http://www.reactome.org/cgi-bin/eventbrowser_st_id?ST_ID=REACT_13638 | 47 | 22 | 10 | 1,28 |
| NFKB_AND_MAP_KINASES_ACTIVATION_MEDIATED_BY_TLR4_SIGNALING_REPERTOIRE | REACT_25281 | http://www.reactome.org/cgi-bin/eventbrowser_st_id?ST_ID=REACT_25281 | 71 | 29 | 11 | 1,28 |
| REGULATION_OF_LIPID_METABOLISM_BY_PEROXISOME_PROLIFERATOR-ACTIVATED_RECEPTOR_ALPHA_(PPARALPHA) | REACT_19241 | http://www.reactome.org/cgi-bin/eventbrowser_st_id?ST_ID=REACT_19241 | 55 | 23 | 10 | 1,28 |
| SIGNALING_BY_ROBO_RECEPTOR | REACT_19351 | http://www.reactome.org/cgi-bin/eventbrowser_st_id?ST_ID=REACT_19351 | 32 | 17 | 11 | 1,29 |
| ACTIVATED_TLR4_SIGNALLING | REACT_6890 | http://www.reactome.org/cgi-bin/eventbrowser_st_id?ST_ID=REACT_6890 | 92 | 34 | 11 | 1,29 |
| PKB-MEDIATED_EVENTS | REACT_456 | http://www.reactome.org/cgi-bin/eventbrowser_st_id?ST_ID=REACT_456 | 28 | 14 | 10 | 1,29 |
| COSTIMULATION_BY_THE_CD28_FAMILY | REACT_19344 | http://www.reactome.org/cgi-bin/eventbrowser_st_id?ST_ID=REACT_19344 | 77 | 29 | 11 | 1,30 |
| TRAF6_MEDIATED_INDUCTION_OF_PROINFLAMMATORY_CYTOKINES | REACT_6782 | http://www.reactome.org/cgi-bin/eventbrowser_st_id?ST_ID=REACT_6782 | 68 | 29 | 11 | 1,33 |
| MAP_KINASE_ACTIVATION_IN_TLR_CASCADE | REACT_21308 | http://www.reactome.org/cgi-bin/eventbrowser_st_id?ST_ID=REACT_21308 | 49 | 25 | 11 | 1,33 |
| CELL_DEATH_SIGNALLING_VIA_NRAGE_NRIF_AND_NADE | REACT_13720 | http://www.reactome.org/cgi-bin/eventbrowser_st_id?ST_ID=REACT_13720 | 64 | 27 | 10 | 1,34 |
| MYD88_MAL_CASCADE_INITIATED_ON_PLASMA_MEMBRANE | REACT_6788 | http://www.reactome.org/cgi-bin/eventbrowser_st_id?ST_ID=REACT_6788 | 87 | 33 | 11 | 1,34 |
| TOLL_LIKE_RECEPTOR_2_(TLR2)_CASCADE | REACT_7980 | http://www.reactome.org/cgi-bin/eventbrowser_st_id?ST_ID=REACT_7980 | 87 | 33 | 11 | 1,34 |
| TOLL_LIKE_RECEPTOR_TLR1_TLR2_CASCADE | REACT_8005 | http://www.reactome.org/cgi-bin/eventbrowser_st_id?ST_ID=REACT_8005 | 87 | 33 | 11 | 1,34 |
| TOLL_LIKE_RECEPTOR_TLR6_TLR2_CASCADE | REACT_8006 | http://www.reactome.org/cgi-bin/eventbrowser_st_id?ST_ID=REACT_8006 | 87 | 33 | 11 | 1,34 |
| INTEGRATION_OF_ENERGY_METABOLISM | REACT_1505 | http://www.reactome.org/cgi-bin/eventbrowser_st_id?ST_ID=REACT_1505 | 125 | 47 | 12 | 1,35 |
| CELL_CYCLE_MITOTIC | REACT_152 | http://www.reactome.org/cgi-bin/eventbrowser_st_id?ST_ID=REACT_152 | 330 | 83 | 11 | 1,37 |
| TRANSMEMBRANE_TRANSPORT_OF_SMALL_MOLECULES | REACT_15518 | http://www.reactome.org/cgi-bin/eventbrowser_st_id?ST_ID=REACT_15518 | 427 | 127 | 12 | 1,38 |
| NEUROTRANSMITTER_RECEPTOR_BINDING_AND_DOWNSTREAM_TRANSMISSION_IN_THE_POSTSYNAPTIC_CELL | REACT_15370 | http://www.reactome.org/cgi-bin/eventbrowser_st_id?ST_ID=REACT_15370 | 136 | 53 | 12 | 1,40 |
| MITOTIC_G1-G1_S_PHASES | REACT_21267 | http://www.reactome.org/cgi-bin/eventbrowser_st_id?ST_ID=REACT_21267 | 135 | 36 | 11 | 1,41 |
| PLATELET_HOMEOSTASIS | REACT_23876 | http://www.reactome.org/cgi-bin/eventbrowser_st_id?ST_ID=REACT_23876 | 81 | 33 | 12 | 1,42 |
| MYD88_CASCADE_INITIATED_ON_PLASMA_MEMBRANE | REACT_27215 | http://www.reactome.org/cgi-bin/eventbrowser_st_id?ST_ID=REACT_27215 | 82 | 33 | 11 | 1,43 |
| TOLL_LIKE_RECEPTOR_10_(TLR10)_CASCADE | REACT_9027 | http://www.reactome.org/cgi-bin/eventbrowser_st_id?ST_ID=REACT_9027 | 82 | 33 | 11 | 1,43 |
| TOLL_LIKE_RECEPTOR_5_(TLR5)_CASCADE | REACT_9061 | http://www.reactome.org/cgi-bin/eventbrowser_st_id?ST_ID=REACT_9061 | 82 | 33 | 11 | 1,43 |
| CYTOKINE_SIGNALING_IN_IMMUNE_SYSTEM | REACT_75790 | http://www.reactome.org/cgi-bin/eventbrowser_st_id?ST_ID=REACT_75790 | 220 | 69 | 11 | 1,43 |
| SIGNAL_TRANSDUCTION_BY_L1 | REACT_22272 | http://www.reactome.org/cgi-bin/eventbrowser_st_id?ST_ID=REACT_22272 | 35 | 20 | 10 | 1,44 |
| MYD88_DEPENDENT_CASCADE_INITIATED_ON_ENDOSOME | REACT_25222 | http://www.reactome.org/cgi-bin/eventbrowser_st_id?ST_ID=REACT_25222 | 81 | 33 | 11 | 1,44 |
| TOLL_LIKE_RECEPTOR_7_8_(TLR7_8)_CASCADE | REACT_9020 | http://www.reactome.org/cgi-bin/eventbrowser_st_id?ST_ID=REACT_9020 | 81 | 33 | 11 | 1,44 |
| TRAF6_MEDIATED_INDUCTION_OF_NFKB_AND_MAP_KINASES_UPON_TLR7_8_OR_9_ACTIVATION | REACT_25024 | http://www.reactome.org/cgi-bin/eventbrowser_st_id?ST_ID=REACT_25024 | 80 | 33 | 11 | 1,46 |
| INTRINSIC_PATHWAY_FOR_APOPTOSIS | REACT_964 | http://www.reactome.org/cgi-bin/eventbrowser_st_id?ST_ID=REACT_964 | 30 | 18 | 11 | 1,47 |
| CIRCADIAN_CLOCK | REACT_24941 | http://www.reactome.org/cgi-bin/eventbrowser_st_id?ST_ID=REACT_24941 | 33 | 16 | 10 | 1,48 |
| PI-3K_CASCADE | REACT_21270 | http://www.reactome.org/cgi-bin/eventbrowser_st_id?ST_ID=REACT_21270 | 57 | 22 | 11 | 1,50 |
| MAPK_TARGETS_NUCLEAR_EVENTS_MEDIATED_BY_MAP_KINASES | REACT_21328 | http://www.reactome.org/cgi-bin/eventbrowser_st_id?ST_ID=REACT_21328 | 30 | 20 | 11 | 1,50 |
| PLATELET_ACTIVATION_SIGNALING_AND_AGGREGATION | REACT_798 | http://www.reactome.org/cgi-bin/eventbrowser_st_id?ST_ID=REACT_798 | 205 | 73 | 11 | 1,55 |
| G_ALPHA_(12_13)_SIGNALLING_EVENTS | REACT_18407 | http://www.reactome.org/cgi-bin/eventbrowser_st_id?ST_ID=REACT_18407 | 77 | 38 | 11 | 1,60 |
| TOLL_LIKE_RECEPTOR_9_(TLR9)_CASCADE | REACT_9047 | http://www.reactome.org/cgi-bin/eventbrowser_st_id?ST_ID=REACT_9047 | 85 | 35 | 11 | 1,61 |
| CD28_CO-STIMULATION | REACT_19183 | http://www.reactome.org/cgi-bin/eventbrowser_st_id?ST_ID=REACT_19183 | 31 | 20 | 11 | 1,65 |
| OPIOID_SIGNALLING | REACT_15295 | http://www.reactome.org/cgi-bin/eventbrowser_st_id?ST_ID=REACT_15295 | 80 | 36 | 11 | 1,68 |
| DOWNSTREAM_SIGNAL_TRANSDUCTION | REACT_17025 | http://www.reactome.org/cgi-bin/eventbrowser_st_id?ST_ID=REACT_17025 | 93 | 46 | 11 | 1,79 |
| ANTIGEN_PROCESSING_UBIQUITINATION_PROTEASOME_DEGRADATION | REACT_75842 | http://www.reactome.org/cgi-bin/eventbrowser_st_id?ST_ID=REACT_75842 | 213 | 76 | 11 | 1,79 |
| DOWNSTREAM_SIGNALING_OF_ACTIVATED_FGFR | REACT_21272 | http://www.reactome.org/cgi-bin/eventbrowser_st_id?ST_ID=REACT_21272 | 100 | 41 | 11 | 1,80 |
| CLASS_I_MHC_MEDIATED_ANTIGEN_PROCESSING_PRESENTATION | REACT_75820 | http://www.reactome.org/cgi-bin/eventbrowser_st_id?ST_ID=REACT_75820 | 251 | 86 | 12 | 1,81 |
| SIGNALING_BY_INTERLEUKINS | REACT_22232 | http://www.reactome.org/cgi-bin/eventbrowser_st_id?ST_ID=REACT_22232 | 106 | 47 | 11 | 1,84 |
| SIGNALING_BY_SCF-KIT | REACT_111040 | http://www.reactome.org/cgi-bin/eventbrowser_st_id?ST_ID=REACT_111040 | 78 | 39 | 11 | 1,84 |
| ADAPTIVE_IMMUNE_SYSTEM | REACT_75774 | http://www.reactome.org/cgi-bin/eventbrowser_st_id?ST_ID=REACT_75774 | 482 | 141 | 12 | 1,85 |
| TRANSMISSION_ACROSS_CHEMICAL_SYNAPSES | REACT_13477 | http://www.reactome.org/cgi-bin/eventbrowser_st_id?ST_ID=REACT_13477 | 190 | 81 | 12 | 1,86 |
| FACTORS_INVOLVED_IN_MEGAKARYOCYTE_DEVELOPMENT_AND_PLATELET_PRODUCTION | REACT_24970 | http://www.reactome.org/cgi-bin/eventbrowser_st_id?ST_ID=REACT_24970 | 125 | 47 | 11 | 1,88 |
| MEMBRANE_TRAFFICKING | REACT_11123 | http://www.reactome.org/cgi-bin/eventbrowser_st_id?ST_ID=REACT_11123 | 133 | 60 | 12 | 1,88 |
| NEURONAL_SYSTEM | REACT_13685 | http://www.reactome.org/cgi-bin/eventbrowser_st_id?ST_ID=REACT_13685 | 289 | 112 | 12 | 1,91 |
| SIGNALING_BY_INSULIN_RECEPTOR | REACT_498 | http://www.reactome.org/cgi-bin/eventbrowser_st_id?ST_ID=REACT_498 | 109 | 41 | 11 | 1,92 |
| SIGNALING_BY_PDGF | REACT_16888 | http://www.reactome.org/cgi-bin/eventbrowser_st_id?ST_ID=REACT_16888 | 122 | 57 | 11 | 1,93 |
| PI3K_CASCADE | REACT_976 | http://www.reactome.org/cgi-bin/eventbrowser_st_id?ST_ID=REACT_976 | 70 | 30 | 11 | 1,98 |
| GOLGI_ASSOCIATED_VESICLE_BIOGENESIS | REACT_19400 | http://www.reactome.org/cgi-bin/eventbrowser_st_id?ST_ID=REACT_19400 | 54 | 35 | 11 | 1,99 |
| SIGNALING_BY_EGFR | REACT_9417 | http://www.reactome.org/cgi-bin/eventbrowser_st_id?ST_ID=REACT_9417 | 109 | 50 | 11 | 2,01 |
| NUCLEAR_RECEPTOR_TRANSCRIPTION_PATHWAY | REACT_15525 | http://www.reactome.org/cgi-bin/eventbrowser_st_id?ST_ID=REACT_15525 | 51 | 26 | 11 | 2,04 |
| SIGNALING_BY_FGFR | REACT_9470 | http://www.reactome.org/cgi-bin/eventbrowser_st_id?ST_ID=REACT_9470 | 114 | 50 | 11 | 2,09 |
| RHO_GTPASE_CYCLE | REACT_11051 | http://www.reactome.org/cgi-bin/eventbrowser_st_id?ST_ID=REACT_11051 | 124 | 53 | 12 | 2,09 |
| SIGNALING_BY_RHO_GTPASES | REACT_11044 | http://www.reactome.org/cgi-bin/eventbrowser_st_id?ST_ID=REACT_11044 | 124 | 53 | 12 | 2,09 |
| IRS-MEDIATED_SIGNALLING | REACT_332 | http://www.reactome.org/cgi-bin/eventbrowser_st_id?ST_ID=REACT_332 | 81 | 36 | 11 | 2,11 |
| IRS-RELATED_EVENTS | REACT_762 | http://www.reactome.org/cgi-bin/eventbrowser_st_id?ST_ID=REACT_762 | 81 | 36 | 11 | 2,11 |
| CLATHRIN_DERIVED_VESICLE_BUDDING | REACT_19187 | http://www.reactome.org/cgi-bin/eventbrowser_st_id?ST_ID=REACT_19187 | 61 | 39 | 11 | 2,17 |
| TRANS-GOLGI_NETWORK_VESICLE_BUDDING | REACT_11235 | http://www.reactome.org/cgi-bin/eventbrowser_st_id?ST_ID=REACT_11235 | 61 | 39 | 11 | 2,17 |
| INSULIN_RECEPTOR_SIGNALLING_CASCADE | REACT_1195 | http://www.reactome.org/cgi-bin/eventbrowser_st_id?ST_ID=REACT_1195 | 86 | 38 | 11 | 2,22 |
| INTERACTION_BETWEEN_L1_AND_ANKYRINS | REACT_22266 | http://www.reactome.org/cgi-bin/eventbrowser_st_id?ST_ID=REACT_22266 | 26 | 15 | 11 | 2,26 |
| CYCLIN_D_ASSOCIATED_EVENTS_IN_G1 | REACT_821 | http://www.reactome.org/cgi-bin/eventbrowser_st_id?ST_ID=REACT_821 | 38 | 20 | 10 | 2,46 |
| G1_PHASE | REACT_1590 | http://www.reactome.org/cgi-bin/eventbrowser_st_id?ST_ID=REACT_1590 | 38 | 20 | 10 | 2,46 |
| NGF_SIGNALLING_VIA_TRKA_FROM_THE_PLASMA_MEMBRANE | REACT_12056 | http://www.reactome.org/cgi-bin/eventbrowser_st_id?ST_ID=REACT_12056 | 136 | 68 | 11 | 3,05 |
| HEMOSTASIS | REACT_604 | http://www.reactome.org/cgi-bin/eventbrowser_st_id?ST_ID=REACT_604 | 467 | 165 | 12 | 3,14 |
| SIGNALLING_BY_NGF | REACT_11061 | http://www.reactome.org/cgi-bin/eventbrowser_st_id?ST_ID=REACT_11061 | 221 | 102 | 11 | 3,52 |
| L1CAM_INTERACTIONS | REACT_22205 | http://www.reactome.org/cgi-bin/eventbrowser_st_id?ST_ID=REACT_22205 | 94 | 55 | 11 | 4,07 |
| DEVELOPMENTAL_BIOLOGY | REACT_111045 | http://www.reactome.org/cgi-bin/eventbrowser_st_id?ST_ID=REACT_111045 | 494 | 186 | 12 | 4,28 |
| AXON_GUIDANCE | REACT_18266 | http://www.reactome.org/cgi-bin/eventbrowser_st_id?ST_ID=REACT_18266 | 266 | 131 | 11 | 5,11 |
