# Supplementary material for: Circulating biomarkers at diagnosis correlate with distant metastases of early luminal-like breast cancer
Source: Genes Immun. 2023 Sep 27;24(5):270–9. doi: 10.1038/s41435-023-00220-z (PMC10575765; doi:10.1038/s41435-023-00220-z)
Supplement: Supplementary file 5 — Supplementary Table S5: Pathway ranking summary of the pathway analysis from the REACTOME software. [file 41435_2023_220_MOESM5_ESM.docx]

**Supplementary Table S5**

*Supplementary Table S5: Pathway ranking summary of the pathway analysis from the REACTOME software. The represented pathways are analyzed from the miRNAs highly expressed in early-metastasizing luminal breast cancer patients. The score represents the alignment of multiple miRNAs involved in pathways in Homo Sapiens. The higher the score, the better the alignment of the multiple microRNAs with that particular pathway.*

| **TERM** | **TERM_ID** | **URL** | **TOTAL_GENES_OF_THE_TERM** | **UNION_TARGETS_IN_THE_TERM** | **MIRS_IN_THE_TERM** | **SCORE** |
| --- | --- | --- | --- | --- | --- | --- |
| METABOLISM_OF_AMINO_ACIDS_AND_DERIVATIVES | REACT_13 | http://www.reactome.org/cgi-bin/eventbrowser_st_id?ST_ID=REACT_13 | 174 | 8 | 5 | 0,51 |
| G_ALPHA_(S)_SIGNALLING_EVENTS | REACT_19327 | http://www.reactome.org/cgi-bin/eventbrowser_st_id?ST_ID=REACT_19327 | 125 | 8 | 5 | 0,55 |
| DIABETES_PATHWAYS | REACT_15380 | http://www.reactome.org/cgi-bin/eventbrowser_st_id?ST_ID=REACT_15380 | 229 | 16 | 5 | 0,58 |
| INTEGRIN_CELL_SURFACE_INTERACTIONS | REACT_13552 | http://www.reactome.org/cgi-bin/eventbrowser_st_id?ST_ID=REACT_13552 | 85 | 10 | 5 | 0,62 |
| TRANSCRIPTION | REACT_1788 | http://www.reactome.org/cgi-bin/eventbrowser_st_id?ST_ID=REACT_1788 | 177 | 10 | 5 | 0,65 |
| HOST_INTERACTIONS_OF_HIV_FACTORS | REACT_6288 | http://www.reactome.org/cgi-bin/eventbrowser_st_id?ST_ID=REACT_6288 | 135 | 14 | 5 | 0,66 |
| MITOTIC_G1-G1_S_PHASES | REACT_21267 | http://www.reactome.org/cgi-bin/eventbrowser_st_id?ST_ID=REACT_21267 | 135 | 12 | 5 | 0,66 |
| SLC-MEDIATED_TRANSMEMBRANE_TRANSPORT | REACT_19118 | http://www.reactome.org/cgi-bin/eventbrowser_st_id?ST_ID=REACT_19118 | 250 | 20 | 5 | 0,67 |
| G0_AND_EARLY_G1 | REACT_111214 | http://www.reactome.org/cgi-bin/eventbrowser_st_id?ST_ID=REACT_111214 | 25 | 6 | 5 | 0,67 |
| MITOTIC_M-M_G1_PHASES | REACT_21300 | http://www.reactome.org/cgi-bin/eventbrowser_st_id?ST_ID=REACT_21300 | 178 | 14 | 5 | 0,68 |
| APOPTOSIS | REACT_578 | http://www.reactome.org/cgi-bin/eventbrowser_st_id?ST_ID=REACT_578 | 148 | 13 | 5 | 0,68 |
| METABOLISM_OF_MRNA | REACT_20605 | http://www.reactome.org/cgi-bin/eventbrowser_st_id?ST_ID=REACT_20605 | 218 | 17 | 5 | 0,69 |
| M_PHASE | REACT_910 | http://www.reactome.org/cgi-bin/eventbrowser_st_id?ST_ID=REACT_910 | 96 | 10 | 5 | 0,70 |
| INTERLEUKIN-1_SIGNALING | REACT_22442 | http://www.reactome.org/cgi-bin/eventbrowser_st_id?ST_ID=REACT_22442 | 40 | 8 | 5 | 0,70 |
| MITOTIC_PROMETAPHASE | REACT_682 | http://www.reactome.org/cgi-bin/eventbrowser_st_id?ST_ID=REACT_682 | 92 | 10 | 5 | 0,71 |
| RESPONSE_TO_ELEVATED_PLATELET_CYTOSOLIC_CA2+ | REACT_1280 | http://www.reactome.org/cgi-bin/eventbrowser_st_id?ST_ID=REACT_1280 | 83 | 10 | 5 | 0,72 |
| THE_ROLE_OF_NEF_IN_HIV-1_REPLICATION_AND_DISEASE_PATHOGENESIS | REACT_6835 | http://www.reactome.org/cgi-bin/eventbrowser_st_id?ST_ID=REACT_6835 | 29 | 7 | 5 | 0,73 |
| DNA_REPLICATION | REACT_383 | http://www.reactome.org/cgi-bin/eventbrowser_st_id?ST_ID=REACT_383 | 200 | 16 | 5 | 0,75 |
| CLASS_A_1_(RHODOPSIN-LIKE_RECEPTORS) | REACT_14828 | http://www.reactome.org/cgi-bin/eventbrowser_st_id?ST_ID=REACT_14828 | 305 | 11 | 5 | 0,75 |
| METABOLISM_OF_RNA | REACT_21257 | http://www.reactome.org/cgi-bin/eventbrowser_st_id?ST_ID=REACT_21257 | 264 | 22 | 5 | 0,75 |
| METABOLISM_OF_PROTEINS | REACT_17015 | http://www.reactome.org/cgi-bin/eventbrowser_st_id?ST_ID=REACT_17015 | 296 | 10 | 5 | 0,76 |
| FACTORS_INVOLVED_IN_MEGAKARYOCYTE_DEVELOPMENT_AND_PLATELET_PRODUCTION | REACT_24970 | http://www.reactome.org/cgi-bin/eventbrowser_st_id?ST_ID=REACT_24970 | 125 | 13 | 5 | 0,78 |
| MUSCLE_CONTRACTION | REACT_17044 | http://www.reactome.org/cgi-bin/eventbrowser_st_id?ST_ID=REACT_17044 | 49 | 8 | 5 | 0,78 |
| CELL-CELL_COMMUNICATION | REACT_111155 | http://www.reactome.org/cgi-bin/eventbrowser_st_id?ST_ID=REACT_111155 | 129 | 14 | 5 | 0,80 |
| METABOLISM_OF_LIPIDS_AND_LIPOPROTEINS | REACT_22258 | http://www.reactome.org/cgi-bin/eventbrowser_st_id?ST_ID=REACT_22258 | 292 | 24 | 5 | 0,80 |
| GPCR_LIGAND_BINDING | REACT_21340 | http://www.reactome.org/cgi-bin/eventbrowser_st_id?ST_ID=REACT_21340 | 410 | 21 | 5 | 0,80 |
| CELL_CYCLE_MITOTIC | REACT_152 | http://www.reactome.org/cgi-bin/eventbrowser_st_id?ST_ID=REACT_152 | 330 | 26 | 5 | 0,81 |
| NETRIN-1_SIGNALING | REACT_22237 | http://www.reactome.org/cgi-bin/eventbrowser_st_id?ST_ID=REACT_22237 | 42 | 7 | 5 | 0,82 |
| HIV_INFECTION | REACT_6185 | http://www.reactome.org/cgi-bin/eventbrowser_st_id?ST_ID=REACT_6185 | 200 | 20 | 5 | 0,86 |
| INTERLEUKIN-2_SIGNALING | REACT_27283 | http://www.reactome.org/cgi-bin/eventbrowser_st_id?ST_ID=REACT_27283 | 42 | 7 | 5 | 0,89 |
| MEMBRANE_TRAFFICKING | REACT_11123 | http://www.reactome.org/cgi-bin/eventbrowser_st_id?ST_ID=REACT_11123 | 133 | 17 | 5 | 0,90 |
| SIGNALLING_TO_RAS | REACT_12033 | http://www.reactome.org/cgi-bin/eventbrowser_st_id?ST_ID=REACT_12033 | 27 | 6 | 5 | 0,91 |
| COSTIMULATION_BY_THE_CD28_FAMILY | REACT_19344 | http://www.reactome.org/cgi-bin/eventbrowser_st_id?ST_ID=REACT_19344 | 77 | 11 | 5 | 0,92 |
| ANTIGEN_PROCESSING_UBIQUITINATION_PROTEASOME_DEGRADATION | REACT_75842 | http://www.reactome.org/cgi-bin/eventbrowser_st_id?ST_ID=REACT_75842 | 213 | 19 | 5 | 0,93 |
| INNATE_IMMUNE_SYSTEM | REACT_6802 | http://www.reactome.org/cgi-bin/eventbrowser_st_id?ST_ID=REACT_6802 | 262 | 21 | 5 | 0,93 |
| CLASS_I_MHC_MEDIATED_ANTIGEN_PROCESSING_PRESENTATION | REACT_75820 | http://www.reactome.org/cgi-bin/eventbrowser_st_id?ST_ID=REACT_75820 | 251 | 22 | 5 | 0,95 |
| OPIOID_SIGNALLING | REACT_15295 | http://www.reactome.org/cgi-bin/eventbrowser_st_id?ST_ID=REACT_15295 | 80 | 12 | 5 | 0,98 |
| GLUTAMATE_BINDING_ACTIVATION_OF_AMPA_RECEPTORS_AND_SYNAPTIC_PLASTICITY | REACT_18347 | http://www.reactome.org/cgi-bin/eventbrowser_st_id?ST_ID=REACT_18347 | 30 | 8 | 5 | 1,10 |
| TRAFFICKING_OF_AMPA_RECEPTORS | REACT_18307 | http://www.reactome.org/cgi-bin/eventbrowser_st_id?ST_ID=REACT_18307 | 30 | 8 | 5 | 1,10 |
| TOLL_RECEPTOR_CASCADES | REACT_6966 | http://www.reactome.org/cgi-bin/eventbrowser_st_id?ST_ID=REACT_6966 | 108 | 16 | 5 | 1,11 |
| TOLL_LIKE_RECEPTOR_9_(TLR9)_CASCADE | REACT_9047 | http://www.reactome.org/cgi-bin/eventbrowser_st_id?ST_ID=REACT_9047 | 85 | 14 | 5 | 1,11 |
| SIGNALING_BY_INSULIN_RECEPTOR | REACT_498 | http://www.reactome.org/cgi-bin/eventbrowser_st_id?ST_ID=REACT_498 | 109 | 14 | 5 | 1,11 |
| TRANSMEMBRANE_TRANSPORT_OF_SMALL_MOLECULES | REACT_15518 | http://www.reactome.org/cgi-bin/eventbrowser_st_id?ST_ID=REACT_15518 | 427 | 41 | 5 | 1,13 |
| NCAM_SIGNALING_FOR_NEURITE_OUT-GROWTH | REACT_18334 | http://www.reactome.org/cgi-bin/eventbrowser_st_id?ST_ID=REACT_18334 | 70 | 11 | 5 | 1,13 |
| ADAPTIVE_IMMUNE_SYSTEM | REACT_75774 | http://www.reactome.org/cgi-bin/eventbrowser_st_id?ST_ID=REACT_75774 | 482 | 44 | 5 | 1,14 |
| MYD88_DEPENDENT_CASCADE_INITIATED_ON_ENDOSOME | REACT_25222 | http://www.reactome.org/cgi-bin/eventbrowser_st_id?ST_ID=REACT_25222 | 81 | 14 | 5 | 1,15 |
| TOLL_LIKE_RECEPTOR_7_8_(TLR7_8)_CASCADE | REACT_9020 | http://www.reactome.org/cgi-bin/eventbrowser_st_id?ST_ID=REACT_9020 | 81 | 14 | 5 | 1,15 |
| INSULIN_RECEPTOR_SIGNALLING_CASCADE | REACT_1195 | http://www.reactome.org/cgi-bin/eventbrowser_st_id?ST_ID=REACT_1195 | 86 | 13 | 5 | 1,15 |
| TRAF6_MEDIATED_INDUCTION_OF_NFKB_AND_MAP_KINASES_UPON_TLR7_8_OR_9_ACTIVATION | REACT_25024 | http://www.reactome.org/cgi-bin/eventbrowser_st_id?ST_ID=REACT_25024 | 80 | 14 | 5 | 1,16 |
| TRAF6_MEDIATED_INDUCTION_OF_PROINFLAMMATORY_CYTOKINES | REACT_6782 | http://www.reactome.org/cgi-bin/eventbrowser_st_id?ST_ID=REACT_6782 | 68 | 13 | 5 | 1,18 |
| MYD88_MAL_CASCADE_INITIATED_ON_PLASMA_MEMBRANE | REACT_6788 | http://www.reactome.org/cgi-bin/eventbrowser_st_id?ST_ID=REACT_6788 | 87 | 15 | 5 | 1,18 |
| TOLL_LIKE_RECEPTOR_2_(TLR2)_CASCADE | REACT_7980 | http://www.reactome.org/cgi-bin/eventbrowser_st_id?ST_ID=REACT_7980 | 87 | 15 | 5 | 1,18 |
| TOLL_LIKE_RECEPTOR_TLR1_TLR2_CASCADE | REACT_8005 | http://www.reactome.org/cgi-bin/eventbrowser_st_id?ST_ID=REACT_8005 | 87 | 15 | 5 | 1,18 |
| TOLL_LIKE_RECEPTOR_TLR6_TLR2_CASCADE | REACT_8006 | http://www.reactome.org/cgi-bin/eventbrowser_st_id?ST_ID=REACT_8006 | 87 | 15 | 5 | 1,18 |
| POTASSIUM_CHANNELS | REACT_75908 | http://www.reactome.org/cgi-bin/eventbrowser_st_id?ST_ID=REACT_75908 | 99 | 12 | 5 | 1,19 |
| NEGATIVE_REGULATION_OF_FGFR_SIGNALING | REACT_111184 | http://www.reactome.org/cgi-bin/eventbrowser_st_id?ST_ID=REACT_111184 | 40 | 8 | 5 | 1,21 |
| IRS-MEDIATED_SIGNALLING | REACT_332 | http://www.reactome.org/cgi-bin/eventbrowser_st_id?ST_ID=REACT_332 | 81 | 13 | 5 | 1,21 |
| IRS-RELATED_EVENTS | REACT_762 | http://www.reactome.org/cgi-bin/eventbrowser_st_id?ST_ID=REACT_762 | 81 | 13 | 5 | 1,21 |
| TOLL_LIKE_RECEPTOR_4_(TLR4)_CASCADE | REACT_6894 | http://www.reactome.org/cgi-bin/eventbrowser_st_id?ST_ID=REACT_6894 | 96 | 16 | 5 | 1,21 |
| TOLL_LIKE_RECEPTOR_3_(TLR3)_CASCADE | REACT_6783 | http://www.reactome.org/cgi-bin/eventbrowser_st_id?ST_ID=REACT_6783 | 74 | 14 | 5 | 1,22 |
| TRIF_MEDIATED_TLR3_SIGNALING | REACT_111135 | http://www.reactome.org/cgi-bin/eventbrowser_st_id?ST_ID=REACT_111135 | 74 | 14 | 5 | 1,22 |
| MYD88_CASCADE_INITIATED_ON_PLASMA_MEMBRANE | REACT_27215 | http://www.reactome.org/cgi-bin/eventbrowser_st_id?ST_ID=REACT_27215 | 82 | 15 | 5 | 1,23 |
| TOLL_LIKE_RECEPTOR_10_(TLR10)_CASCADE | REACT_9027 | http://www.reactome.org/cgi-bin/eventbrowser_st_id?ST_ID=REACT_9027 | 82 | 15 | 5 | 1,23 |
| TOLL_LIKE_RECEPTOR_5_(TLR5)_CASCADE | REACT_9061 | http://www.reactome.org/cgi-bin/eventbrowser_st_id?ST_ID=REACT_9061 | 82 | 15 | 5 | 1,23 |
| ACTIVATED_TLR4_SIGNALLING | REACT_6890 | http://www.reactome.org/cgi-bin/eventbrowser_st_id?ST_ID=REACT_6890 | 92 | 16 | 5 | 1,24 |
| NFKB_AND_MAP_KINASES_ACTIVATION_MEDIATED_BY_TLR4_SIGNALING_REPERTOIRE | REACT_25281 | http://www.reactome.org/cgi-bin/eventbrowser_st_id?ST_ID=REACT_25281 | 71 | 14 | 5 | 1,25 |
| NEUROTRANSMITTER_RELEASE_CYCLE | REACT_13723 | http://www.reactome.org/cgi-bin/eventbrowser_st_id?ST_ID=REACT_13723 | 36 | 10 | 5 | 1,26 |
| NEUROTRANSMITTER_RECEPTOR_BINDING_AND_DOWNSTREAM_TRANSMISSION_IN_THE_POSTSYNAPTIC_CELL | REACT_15370 | http://www.reactome.org/cgi-bin/eventbrowser_st_id?ST_ID=REACT_15370 | 136 | 21 | 5 | 1,28 |
| CYTOKINE_SIGNALING_IN_IMMUNE_SYSTEM | REACT_75790 | http://www.reactome.org/cgi-bin/eventbrowser_st_id?ST_ID=REACT_75790 | 220 | 30 | 5 | 1,29 |
| SIGNALLING_TO_ERKS | REACT_12058 | http://www.reactome.org/cgi-bin/eventbrowser_st_id?ST_ID=REACT_12058 | 35 | 8 | 5 | 1,31 |
| MYD88-INDEPENDENT_CASCADE_INITIATED_ON_PLASMA_MEMBRANE | REACT_6809 | http://www.reactome.org/cgi-bin/eventbrowser_st_id?ST_ID=REACT_6809 | 75 | 15 | 5 | 1,33 |
| MAP_KINASE_ACTIVATION_IN_TLR_CASCADE | REACT_21308 | http://www.reactome.org/cgi-bin/eventbrowser_st_id?ST_ID=REACT_21308 | 49 | 12 | 5 | 1,33 |
| PLATELET_ACTIVATION_SIGNALING_AND_AGGREGATION | REACT_798 | http://www.reactome.org/cgi-bin/eventbrowser_st_id?ST_ID=REACT_798 | 205 | 27 | 5 | 1,39 |
| SIGNALING_BY_INTERLEUKINS | REACT_22232 | http://www.reactome.org/cgi-bin/eventbrowser_st_id?ST_ID=REACT_22232 | 106 | 20 | 5 | 1,49 |
| FRS2-MEDIATED_CASCADE | REACT_21247 | http://www.reactome.org/cgi-bin/eventbrowser_st_id?ST_ID=REACT_21247 | 38 | 9 | 5 | 1,52 |
| HEMOSTASIS | REACT_604 | http://www.reactome.org/cgi-bin/eventbrowser_st_id?ST_ID=REACT_604 | 467 | 52 | 5 | 1,62 |
| SIGNALING_BY_SCF-KIT | REACT_111040 | http://www.reactome.org/cgi-bin/eventbrowser_st_id?ST_ID=REACT_111040 | 78 | 17 | 5 | 1,80 |
| L1CAM_INTERACTIONS | REACT_22205 | http://www.reactome.org/cgi-bin/eventbrowser_st_id?ST_ID=REACT_22205 | 94 | 22 | 5 | 1,91 |
| TRANSMISSION_ACROSS_CHEMICAL_SYNAPSES | REACT_13477 | http://www.reactome.org/cgi-bin/eventbrowser_st_id?ST_ID=REACT_13477 | 190 | 33 | 5 | 1,96 |
| SIGNALING_BY_PDGF | REACT_16888 | http://www.reactome.org/cgi-bin/eventbrowser_st_id?ST_ID=REACT_16888 | 122 | 25 | 5 | 2,02 |
| DOWNSTREAM_SIGNAL_TRANSDUCTION | REACT_17025 | http://www.reactome.org/cgi-bin/eventbrowser_st_id?ST_ID=REACT_17025 | 93 | 20 | 5 | 2,04 |
| SIGNALING_BY_EGFR | REACT_9417 | http://www.reactome.org/cgi-bin/eventbrowser_st_id?ST_ID=REACT_9417 | 109 | 25 | 5 | 2,17 |
| DOWNSTREAM_SIGNALING_OF_ACTIVATED_FGFR | REACT_21272 | http://www.reactome.org/cgi-bin/eventbrowser_st_id?ST_ID=REACT_21272 | 100 | 23 | 5 | 2,30 |
| SIGNALING_BY_FGFR | REACT_9470 | http://www.reactome.org/cgi-bin/eventbrowser_st_id?ST_ID=REACT_9470 | 114 | 26 | 5 | 2,40 |
| NEURONAL_SYSTEM | REACT_13685 | http://www.reactome.org/cgi-bin/eventbrowser_st_id?ST_ID=REACT_13685 | 289 | 44 | 5 | 2,41 |
| SIGNALLING_BY_NGF | REACT_11061 | http://www.reactome.org/cgi-bin/eventbrowser_st_id?ST_ID=REACT_11061 | 221 | 45 | 5 | 3,05 |
| NGF_SIGNALLING_VIA_TRKA_FROM_THE_PLASMA_MEMBRANE | REACT_12056 | http://www.reactome.org/cgi-bin/eventbrowser_st_id?ST_ID=REACT_12056 | 136 | 32 | 5 | 3,10 |
| DEVELOPMENTAL_BIOLOGY | REACT_111045 | http://www.reactome.org/cgi-bin/eventbrowser_st_id?ST_ID=REACT_111045 | 494 | 76 | 5 | 3,22 |
| AXON_GUIDANCE | REACT_18266 | http://www.reactome.org/cgi-bin/eventbrowser_st_id?ST_ID=REACT_18266 | 266 | 52 | 5 | 3,27 |
